# *TREM2* in Neurodegenerative Disorders: Mutation Spectrum, Pathophysiology, and Therapeutic Targeting

**DOI:** 10.3390/ijms26157057

**Published:** 2025-07-22

**Authors:** Hyewon Yang, Danyeong Kim, YoungSoon Yang, Eva Bagyinszky, Seong Soo A. An

**Affiliations:** 1 Department of Bionano Technology, Gachon University, Seongnam 13120, Republic of Korea; yhw0528@gachon.ac.kr (H.Y.); dan627328@gmail.com (D.K.); 2 Department of Neurology, Veterans Medical Research Institute, Veterans Health Service Medical Center, Seoul 05368, Republic of Korea; 3 Department of Neurology, Soonchunhyang University Hospital, Cheonan 31151, Republic of Korea; astro76@naver.com; 4 Department of Industrial and Environmental Engineering, Graduate School of Environment, Gachon University, Seongnam 13120, Republic of Korea

**Keywords:** TREM2, mutation, Alzheimer’s disease, frontotemporal dementia, Nasu-Hakola disease, therapy

## Abstract

TREM2 (triggering receptor expressed on myeloid cells 2) is a membrane-bound receptor primarily expressed on microglia in the central nervous system (CNS). TREM2 plays a crucial role in regulating immune responses, phagocytosis, lipid metabolism, and inflammation. Mutations in the TREM2 gene have been linked to various neurodegenerative diseases, including Alzheimer’s disease (AD), frontotemporal dementia (FTD), Parkinson’s disease (PD), and Nasu–Hakola disease (NHD). These mutations are suggested to impair microglial activation and reduce the ability to clear amyloid aggregates, leading to exacerbated neuroinflammatory responses and accelerating disease progression. This review provides an overview of TREM2 structure, functions, and known pathogenic variants—including Arg47His, Arg62His, His157Tyr, Tyr38Cys, and Thr66Met. Furthermore, the molecular and cellular consequences of *TREM2* mutations are introduced, such as impaired ligand binding, altered protein folding and trafficking, enhanced TREM2 shedding, and dysregulated inflammatory signaling. We also highlight recent advances in therapeutic strategies aimed at modulating TREM2 signaling. These include monoclonal antibodies (e.g., AL002, CGX101), small molecule agonists, and gene/cell-based therapies that seek to restore microglial homeostasis, enhance phagocytosis, and reduce neuroinflammation. While these approaches show promise in in vivo/in vitro studies, their clinical translation may be challenged by disease heterogeneity and mutation-specific responses. Additionally, determining the appropriate timing and precise dosing will be essential.

## 1. Introduction

Triggering receptor expressed on myeloid cells 2 (TREM2) is a membrane glycoprotein that plays an important role in the survival, activation, and regulation of microglia phagocytosis. Furthermore, *TREM2* is involved in the inflammatory response to brain homeostasis, damage, or neurodegeneration [1,2,3]. TREM2-encoded receptors are associated with innate immune responses and chronic inflammation, and they are essential for microglia to detect and respond to neurodegenerative signals [4,5]. TREM2 can bind to DNAX-activating protein 12 (DAP12) and form a signal complex, leading to the activation of intracellular signaling pathways. TREM2 has been verified as a risk factor for Alzheimer’s disease (AD), especially late-onset AD (LOAD) [2,6].

*TREM2* mutations should be noted as important research subjects, given the strong correlations observed between *TREM2* mutations and AD. Additionally, associations have been found between *TREM2* mutations and other neurodegenerative diseases, such as frontotemporal dementia (FTD), Parkinson’s disease (PD), and Nasu–Hakola disease (NHD) [1,2,3,4,5].

Mutations in Amyloid Precursor Protein (*APP*), Presenilin-1 (*PSEN1*), and Presenilin-2 (*PSEN2*) are the main causative factors for early-onset AD (EOAD) due to their roles in amyloid beta (Aβ) production and aggregation. However, mutations in *APP*, *PSEN1*, and *PSEN2* have been relatively rarely observed in EOAD patients. Recent studies have revealed that additional genetic factors involved in LOAD can also play a role in EOAD. *TREM2* has been suggested as a candidate genetic factor for EOAD [7]. Studies have shown that TREM2 function is closely related to APP processing pathways and downstream pathways in neurodegenerative diseases [7,8]. Unlike *APP* and *PSEN* mutations, which directly affect Aβ production, *TREM2* mutations are suggested to influence AD onset through the clearance of amyloid deposits and neuroinflammatory responses to Aβ aggregates [7,8]. Understanding the interactions between AD-associated genes and *TREM2* is critical for elucidating disease mechanisms and identifying potential therapeutic targets. Although these mutations are rarely reported, they provide important insights into disease mechanisms and highlight their significant roles in the development of AD models.

## 2. TREM2 Gene and TREM2 Protein Structure and Function

TREM2 is a transmembrane protein that functions as a cell surface receptor The *TREM*2 gene is located within the *TREM* gene cluster on chromosome 6p21.1 in humans and on chromosome 17 in mice [9,10,11,12]. Five major *TREM* genes have been identified in humans: *TREM1*, *TREM2*, *TREM3*, *TREML1*, and *TREML2*. In mice, *TREM4* and *TREM5* were identified too. These proteins are known to play an important role in controlling immune response and inflammation. Five coding exons have been identified in the TREM2 gene, which can encode a full-length protein consisting of 230 amino acids. Recent studies have identified several heterozygous or homozygous variants in the TREM2 gene, fwhich may be involved in disease-related mechanisms. The TREM2 protein has been found to be independently expressed in myeloid cells [13,14,15]. TREM2 has also been reported to be expressed in tissue-resident macrophages, dendritic cells, and bone marrow cells, including microglia, which are identified as major innate immune cells in the brain [15,16].

**Figure 1:**
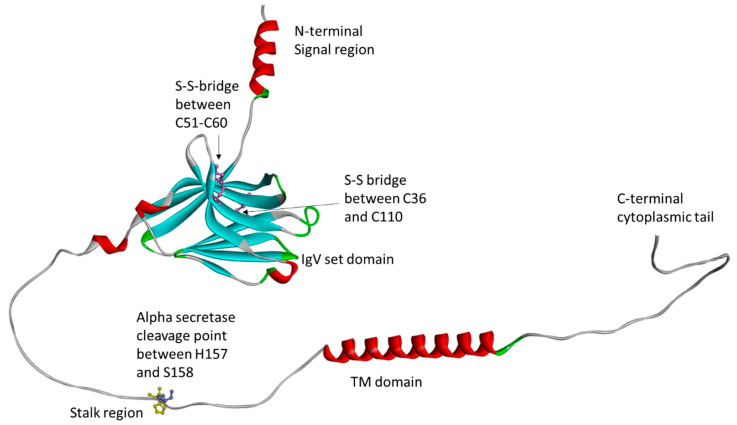
Three-dimensional structure of normal TREM2 protein. This figure was generated by AlphaFold Colab online tool (https://colab.research.google.com/github/sokrypton/ColabFold/blob/main/AlphaFold2.ipynb, accessed on 1 May 2025).

This protein has several domains. A signal peptide sequence is located in the N-terminal region (residues 1–18), followed by the immunoglobulin domain (IgV set domain) between residues 19 and 130. A shorter stalk region is located between residues 131 and 174. Additionally, TREM2 has a transmembrane domain (TM) between residues 175 and 195, followed by a C-terminal cytoplasmic tail between residues 196 and 230. The TREM2 protein has a cleavage site for alpha-secretase at Histidine 157 and Serine 158 residues in the stalk domain. Two disulfide bridges have been identified in TREM2, between cysteine 36 and 110 and cysteine 51 and 60 (Figure 1) [1,2,3,4,5,6,7,8,9,10,17] (https://www.alzforum.org/mutations/trem2, accessed on 1 June 2025).

TREM2 protein is highly expressed in the brain and plays an active role in the activation of microglia in the central nervous system (CNS) homeostasis, suggesting that TREM2 dysfunctions can be significantly involved in AD and other neurodegenerative diseases [7,8,9]. Furthermore, TREM2 has been suggested to regulate the mitochondrial function of microglia, promoting energy production to meet the brain’s energy demands [10,11,12].

TREM2 protein can bind to various ligands on cell surface receptors, activating intracellular signaling pathways. TREM2 contains an essential extracellular immunoglobulin (Ig) domain for binding to different ligands. In particular, the Ig-like domain plays a key role in the ligand binding and immune pathways of TREM2 [18,19]. The Ig-like domain has been verified to bind to associated ligands of cell damage and apoptosis, extracellular plaques, anionic lipids, and Aβ [14]. In addition, the Ig-like domain of TREM2 can be released as soluble TREM2 (sTREM2) through proteolytic cleavage by the metalloproteases ADAM10 or ADAM17 at histidine 157, located in the stalk region. Interestingly, sTREM2 has been suggested to impact immunomodulatory function [19].

In addition, TREM2 can bind to various ligands, including Apolipoprotein E ε4 allele (*APOE*4) and Aβ, thereby regulating microglial functions such as Aβ phagocytosis, transcriptional changes, and their conversion to disease-associated phenotypes [18,19]. TREM2 also plays a significant role in maintaining lipid homeostasis in the brain by controlling lipid metabolism. This reflects that TREM2 can function as a sensor for lipids derived from myelin and initiates intracellular signaling pathways that control lipid metabolism and degradation in microglia [18]. Myelin is a lipoprotein layer that surrounds the axons in the CNS and peripheral nervous system (PNS) and plays an important role in rapid and efficient nerve signal transmission. In NHDs, TREM2 dysfunctions have been found to cause the decline of microglial myelin functions, inducing myelin loss and abnormal axon structures in the brain. This process impairs nerve signaling, leading to neurodegeneration. In addition, TREM2 can interact with lipid metabolism-associated proteins, such as APOE, to regulate brain lipid transport and metabolism. The TREM2 and APOE interaction has been found to regulate the lipid environment around Aβ plaques and enhances microglial Aβ clearance ability [16]. The Aβ-binding ability of TREM2 may vary depending on its interactions with other factors, including APOE, which can also disturb Aβ degradation and impact Aβ-induced microglial depolarization, inflammatory molecule expression, migration, proliferation, apoptosis, or morphological changes [18,19,20].

TREM2 has been reported to inhibit excessive inflammation by regulating inflammatory response. Furthermore, it may contribute to maintaining the normal function of nerve cells by recognizing damage, disease, and various ligands. TREM2 assists microglia in identifying and clearing damaged cells, myelin fragments, and other brain wastes. Additionally, TREM2 controls inflammatory signals involved in the regulation of excessive inflammatory responses and in neural protection [21,22]. TREM2 activation regulates inflammatory response and homeostasis by stimulating the expression of anti-inflammatory cytokines and suppressing pro-inflammatory cytokine production [7,20]. Moreover, Toll-like receptors (TLRs) can detect pathogen-related molecular patterns to trigger inflammatory responses, whereas TREM2 contributes to neuroprotection by regulating these inflammatory processes [19,20,21,22,23,24,25]. Also, axonal loss can disturb synaptic and neuronal connectivity, resulting in neuronal loss, cognitive decline, and the development of dementia [19,20,21].

TREM2 plays a crucial role in slowing the progression of neurodegeneration by controlling microglial activity and functions. Understanding the function and disease involvement of TREM2 is essential. Gaining deeper insight into TREM2 functions is critical and may offer promising avenues for research on neurodegenerative diseases.

## 3. Neurodegenerative Disease and TREM2

**Figure 2:**
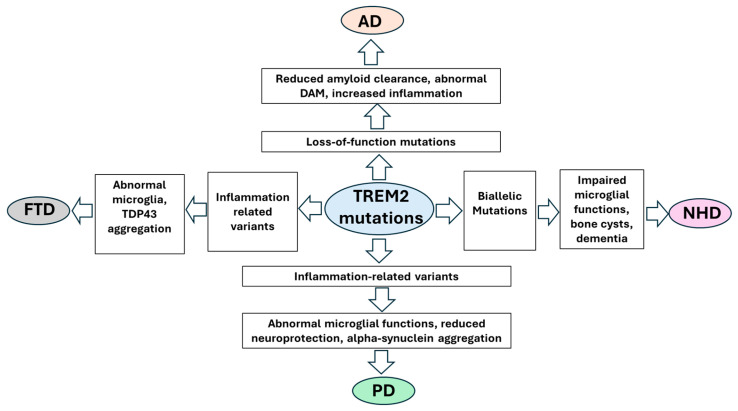
Impact of *TREM2* on AD, FTD, PD, and NHD. *TREM2* mutations were associated with immune dysfunctions, leading to abnormal microglial aggregation and reduced clearance of misfolded proteins, including amyloid peptides, alpha synuclein, or TDP43.

*TREM2* mutations were suggested to impact multiple neurodegenerative diseases, including AD, FTD, NHD, and PD (Figure 2). Furthermore, abnormal *TREM2* expression could impact multiple sclerosis (MS); however, no specific variants could be linked directly to MS onset. *TREM2* gene and protein expression were found to be elevated in MS patients. *TREM2* may impact MS by modulating inflammatory responses and facilitating the clearance of myelin debris, which should be for remyelination [25].

### 3.1. AD and TREM2

Alzheimer’s disease (AD) is the most common form of chronic neurodegenerative disease worldwide that is characterized by the aggregation of Aβ peptide deposits into plaques in the brain. The main phenotypes of AD include memory loss, impaired executive function, personality changes, and the gradual inability to perform daily activities [26]. Multiple pathways can contribute to AD onset, including genetic modification and polymorphism, abnormal immune or inflammatory responses, and environmental factors [27,28].

Elevated *TREM2* expression in the early stages of AD may indicate increased microglial activity against Aβ. Microglia can respond to Aβ plaques by surrounding and clearing them, during which *TREM2* expression and immune response are increased [11]. Alterations in TREM2 expression have been reported in the brains of AD patients, indicating that TREM2 may play a crucial role in AD progression [29,30,31,32]. The reduced microglial phagocytic ability due to decreased TREM2 expression could accelerate AD progression. Upon inhibition of Aβ plaque clearance from ineffective TREM2 activity, subsequent amyloid accumulations in the brain would lead to sustained neuroinflammatory responses and neuronal death, thereby contributing to memory and cognitive decline, leading to AD onset [11].

*APOE*4 has been verified as the strongest genetic risk factor for AD [33]. In particular, the interactions between *TREM2* and *APOE*4 are suggested to play a significant role in regulating phagocytosis and neuroinflammatory pathways that induce the AD pathomechanism [30,31]. The *APOE* gene influences both cerebrospinal fluid (CSF) sTREM2 levels and the AD risk associated with the *APOE*4 variant. Soluble TREM2 (sTREM2) has been verified to be associated with AD progression, and its levels can increase during the early AD process [34,35,36]. In individuals carrying the *APOE* E4 allele, higher cerebrospinal fluid (CSF) sTREM2 levels were prospectively correlated with reduced risks of AD, cognitive decline, and neurodegenerative diseases associated with *APOE*4 [31,35]. In particular, sTREM2 levels in CSF may play a role in reducing the risk of *APOE*4-related AD [29,32]. The *APOE* E4 allele along with *TREM2* mutations (including *TREM2* Arg47His) were associated with a higher degree of cognitive dysfunction, as they could impair microglial phagocytosis, leading to reduced amyloid clearance and a stronger degree of inflammation [31,32,33,34,35,36,37,38].

**Figure 3:**
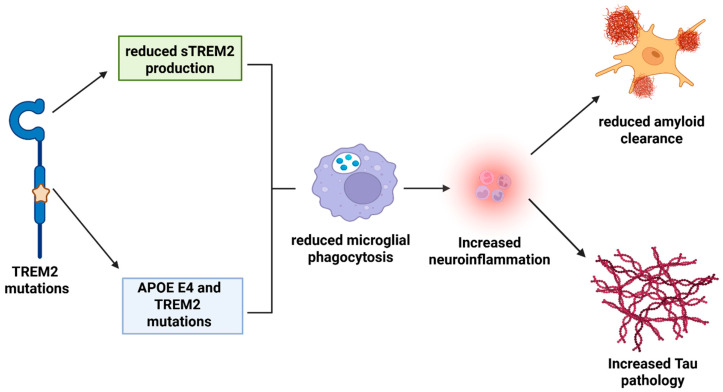
*TREM2* mutations and their possible pathogenic mechanisms in AD.

Mutations in *TREM2* have been associated with reduced CSF sTREM2 levels, leading to impaired Aβ clearance ability by the microglia [32,39]. Additionally, higher levels of CSF sTREM2 in AD patients may indicate the microglial response to AD pathological changes [39]. Conversely, reduced TREM2 levels were associated with neurofibrillary tangle degeneration, cognitive impairment, and activation of inflammatory responses [36,37,38,39,40,41,42,43,44,45]. On the other hand, higher sTREM2 levels are suggested to reflect a higher degree of microglia activation, leading to reduced inflammatory responses and neurodegenerative processes in the brain [40,41,42,43,44,45], which explains the consequences of genetic variation in TREM2 affecting patients with AD by regulating immune responses. Figure 3 summarizes the probable pathogenic mechanisms of *TREM2* in AD. *TREM2* mutations may be associated with reduced microglial phagocytosis and elevated degree of neuroinflammation, leading to reduced amyloid clearance and increased Tau pathology. In the presence of *APOE* E4 allele, effects of *TREM2* mutations and may result in more severe disease phenotype.

### 3.2. TREM2 and NHD

The homozygous loss-of-function mutations of *TREM2* have been verified to play a role in an autosomal recessive disease called Nasu–Hakola disease (NHD), which affects the brain and bones [45,46,47]. NHD patients usually present with disease symptoms at a relatively young age. In most cases, the disease can begin with a bone cyst, leading to pain, swelling, and fractures in the wrist and ankle in their 30 s, followed by early-onset dementia in their 40 s [48]. Main symptoms of NHD include personality changes, behavioral impairment, or language dysfunctions. Memory decline may also be possible, but it tends to be less pronounced compared to that in Alzheimer’s disease (AD) patients. At least 11 *TREM2* mutations have been associated with NHD, including Tyr38Cys, Trp50Cys, Thr66Met, and Va126Gly [45,46,47,48,49,50].

Mutations in the *TREM2* and DAP12 genes are associated with NHD [40,44,45]. TREM2 and DAP12 can interact, regulating the proliferation and survival of osteoclast precursors, which are involved in bone resorption and in promoting bone resorption activity in osteoclasts [51,52,53,54,55,56,57,58]. Since TREM2 and DAP12 are specifically expressed in myeloid cells, NHD can be attributed to impaired functions of microglia and osteoclasts.

**Figure 4:**
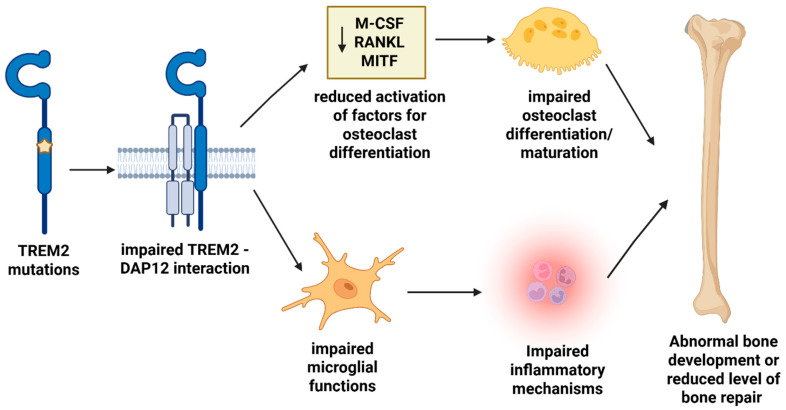
Involvement of *TREM2* in bone repair mechanisms.

Osteoclasts are formed when human blood monocytes differentiate in response to specific proteins, including Macrophage Colony-Stimulating Factor (M-CSF), Microphthalmia-associated Transcription Factor (MITF), Receptor Activator of Nuclear Factor κB Ligands (RANKL), or NF-κB. However, such differentiations in cells of NHD patients with impaired DAP12 or TREM2 functions are not properly performed and may result in cytoskeletal dysfunctions. These pathways can lead to reduced bone resorption capacity, which contributes to bone lesion formation in NHD patients [56,57]. Figure 4 summarizes the bone impairment-related mechanisms of *TREM2* mutations in NHD.

Additionally, signals from the TREM2-DAP12 complex play a key role in driving microglia transition from a homeostatic state to one associated with neurodegenerative diseases [57]. The expression levels of the *TREM2* and DAP12 genes may be variable in microglia in NHD patients. The loss-of-function mutations in TREM2 affect the central nervous system (CNS) in patients with Nasu–Hakola disease (NHD). These findings demonstrate the importance of understanding the underlying mechanisms of dementia and protein impairment in NHD [43,57,58]. TREM2 and DAP12 deficiency can cause excessive inflammatory microglia activation, leading to brain damage with Aβ plaques and affecting damaged neurons [11,58]. It also exacerbates the pro-inflammatory activation of microglia against a variety of stimuli.

### 3.3. TREM2 and FTD

Frontotemporal dementia (FTD) is a neurodegenerative disease that primarily affects the atrophic frontal and temporal lobes, unlike other neurodegenerative diseases such as Alzheimer’s disease (AD), Parkinson’s disease (PD), and dementia with Lewy bodies (DLB). The main clinical phenotypes of FTD can include personality changes, behavioral changes, and language impairment, though memory decline cannot be entirely ruled out. Both genetic and environmental factors have been verified to impact the disease’s onset. Heterozygous expressions of the *TREM2* mutation have been associated with FTD [59,60,61,62], which is consistent with an increased risk in AD and PD [62]. The specific mutation, His157Tyr in *TREM2*, in particular, has been verified in connection with the clinical phenotype of FTD, as carrier patients presented with symptoms of behavioral changes and cognitive decline. Additionally, *TREM2* has been verified to play a role in regulating the innate immune system and microglial functions within the brain, with multiple pathways potentially contributing to the development of FTD [59,60,61,62,63,64].

Although both *TREM2* and TAR DNA-binding protein 43 (TDP-43) have been suggested to be significant factors in FTD, *TREM2* has been verified as a genetic variation in FTD, as TREM2 mutations can disturb microglial function, leading to disease progression. Furthermore, TREM2 could be a therapeutic target for FTD [65,66,67,68].

*TREM2* has been verified to act as a microglial mediator that interacts with TDP-43 to confer neuroprotection and is involved in regulating TDP-43 accumulation [65,66,67,68]. TDP-43 is an RNA-binding protein that has been verified as a genetic risk factor and pathologic hallmark of FTD [69]. Impaired TDP-43 function can induce exon skipping, thereby impairing cellular function. In TDP-43 proteinopathy, TREM2 has been suggested to be significantly involved in the regulation of phosphorylated and ubiquitinated TDP-43 aggregation and is associated with cleaved TDP-43 fragments, which can exacerbate neurotoxicity [70]. Upon TREM2 binding to TDP-43, microglial phagocytosis is promoted, which may facilitate TDP-43 elimination and attenuate neuronal damage [65]. Without proper TREM2 function, microglial activity can be reduced, thereby resulting in a diminished capacity to effectively clear the neurotoxic TDP-43 aggregates. This process exacerbates neuronal damage and may contribute to motor impairment. Furthermore, loss of TREM2 function may result in the accumulation of neurotoxic TDP-43, which precipitates neurodegeneration, and TDP-43 proteins exhibit an increased tendency to become hyperphosphorylated and aggregate [65,71].

### 3.4. TREM2 and PD

Parkinson’s disease (PD) is a neurodegenerative disease characterized by the degeneration of dopaminergic neurons. As a potential candidate gene for PD, *TREM2* may influence both the risk and progression of the disease while offering neuroprotective effects through its regulation of microglial activity [72,73]. TREM2 can interact with Transmembrane Protein 59 (TMEM59), a membrane-bound protein, to enhance autophagy, which may protect dopaminergic neurons against inflammation-associated damage in Parkinson’s disease [74,75]. TREM2 can also protect dopamine neurons by targeting Nod-like receptor pyrin domain-containing protein 3 (NLRP3), a protein within the pro-inflammatory cytokine complex, by inhibiting microglial activation [73,76]. *TREM2* gene mutations, including Arg47His, have been observed to increase PD risk. Impaired TREM2 functions have been shown to induce neurodegeneration and neuroinflammatory processes in PD models [52,77,78].

Interaction between TREM2 and Unc-51-like autophagy-activating kinase 1 (ULK1) promotes autophagy within microglia, which confers a neuroprotective effect in the inflammatory environment of PD. Alpha-melanocyte-stimulating hormone (α-MSH) is a peptide hormone derived from a pro-opiomelanocortin (POMC) gene and performs various physiological functions, including energy homeostasis. Although increased α-MSH in the cerebrospinal fluid (CSF) of PD patients has been suggested to impair cell autophagy and induce the accumulation of α-synuclein, TREM2 is thought to contribute to improving this autophagy dysfunction [76,79].

In addition, TREM2 participates in controlling α-synuclein-induced neurodegeneration and neuroinflammation [80,81]. Deficiency of TREM2 has been found to enhance α-synuclein-induced inflammatory responses and further accelerate the loss of dopaminergic neurons in PD models. An exacerbation of dopaminergic neuronal loss was observed in *TREM2*-deficient mouse models, indicating that TREM2 may play a significant role in neuroprotection in PD [82,83,84,85]. The loss of *TREM2* results in reduced phagocytosis by microglia, leading to the accumulation of α-synuclein. Furthermore, reduced TREM2 levels result in elevated levels of TLR4 immune protein, leading to an increased degree of inflammation and inhibition of α-synuclein fiber phagocytosis. Abnormal TREM2 signaling has been shown to worsen the inflammatory response of microglia to α-synuclein and increase α-synuclein-related neurodegeneration [83]. Therefore, maintaining proper TREM2 functions is essential in inhibiting PD progression and protecting dopaminergic neurons [82,83,84].

## 4. TREM2 Pathological Mechanism

**Figure 5:**
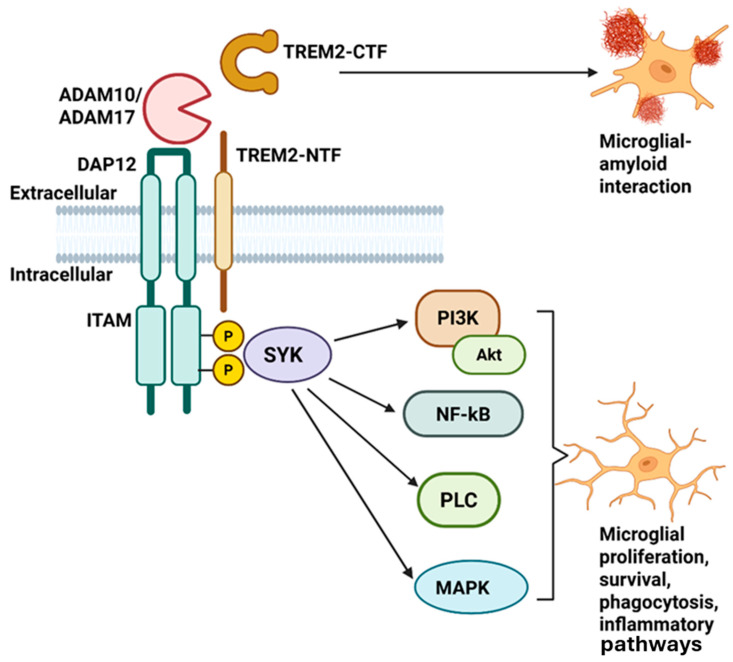
TREM2 signaling pathway.

TREM2 can form a complex with DAP12 for signal transmission [86]. Ligand binding to TREM2 can result in the activation of the TREM2-DAP12 complex, leading to Immunoreceptor Tyrosine-based Activation Motif (ITAM) phosphorylation in the intracellular domain of DAP12. This, in turn, leads to SYK kinase activation and the regulation of signaling pathways, including extracellular nuclear factor kappa-light-chain-enhancer of activated B cells (NFκB), phosphatidylinositol 3-kinase (PI3K), Mitogen-Activated Protein Kinase (MAPK), and phospholipase-c (PLCγ) signaling. These pathways impact cell proliferation, survival, or inflammation. DAP12 can stabilize the C-terminal fragment of TREM2 (TREM2-CTF) and regulate the inflammatory response and TREM2-Aβ interaction, leading to cytokine expression and release (Figure 5) [22,87].

Microglial activation in cases of TREM2 mutations results in an increased release of pro-inflammatory cytokines, leading to an inflammatory environment within the brain [17,18,19]. Pro-inflammatory cytokines have been verified to promote Tau protein phosphorylation, leading to neurofibrillary tangle (NFT) formation and exacerbating nerve cell damage [86,87,88,89,90,91,92]. Chronic neuroinflammation can impair nerve cell functions and accelerate AD progression. Suppression of TREM2 expression can increase the production of pro-inflammatory cytokines, including TNF-α, IL-1β, and IL-6, and promote neuroinflammation [93]. Furthermore, suppression of the NF-κB signaling pathway can inhibit the release of inflammatory factors [94]. Chronic brain inflammation can directly harm nerve cells by triggering microglia to continuously produce pro-inflammatory cytokines and reactive oxygen species (ROS). These inflammatory mediators can lead to synaptic dysfunctions, excitotoxicity, and oxidative stress-related damage, resulting in decreased neuronal function and loss of nerve cells [90,91]. By increasing the activity of amyloid-producing enzymes and suppressing Aβ clearance, the inflammatory environment promotes Aβ plaque formation [92,95,96]. This process can inhibit homeostasis maintenance and suppress nerve cell regeneration, accelerating the progression of neurodegeneration [97,98]. Particularly, since amyloid-induced neurotoxicity can depend on CD36/TLR4/TLR6-mediated inflammatory pathways, chronic inflammation induced by TREM2 mutations can directly increase neurotoxicity, which may accelerate AD-related neurodegeneration. Thus, TREM2 is suggested as a crucial factor in the pathological mechanisms of AD [14,97,98].

The microglia cells were verified as a resident immune cell in the CNS, and they could exhibit diverse phenotypes and functions. Microglia could play a crucial role in maintaining brain homeostasis and protecting against the pathology. They were verified to have morphological plasticity, which could allow for them to adapt easily the microenvironment and change their phenotypes quickly in case of disturbances. Due to the traditional classification, microglia has two subtypes: M1 (pro-inflammatory) and M2 (anti-inflammatory) phenotypes. However, this classification seemed to be oversimplified. In reality, the microglia could exhibit a continuous spectrum of activation phenotypes, and mixed phenotypes, which could express both M1 and M2 markers, especially in case of aging and pathological conditions [99,100]. Disease-associated microglia (DAM) was verified as a distinct subtype of microglia that appeared in different stages of neurodegenerative diseases. DAM activation is a two-stage process: the first stage is a TREM2-independent step, where homeostatic microglia could form to Stage 1 DAM. The second stage, where the transformation from Stage 1 DAM to Stage 2 DAM occurs, was verified to be dependent on the TREM2 signaling. In this stage, genes related to lipid metabolism and phagocytosis were upregulated [101,102]. In the DAM1 stage, several genes are activated, including Tyrobp, Apoe, and B2m. Furthermore, several microglial checkpoint genes could be downregulated, such as Cx3cr1 and P2ry12/P2ry13. In the TREM2-dependent DAM2 stage, phagocytic and lipid metabolism-related genes, including Cst7, Lpl, and CD9, could be upregulated [103,104].

DAM has been found to accumulate around amyloid plaques. Furthermore, DAM has been suggested to play a role in amyloid-beta buildup and in Tau phosphorylation. Their activation follows a two-step process involving an initial TREM2-independent phase followed by a TREM2-dependent activation phase [105,106,107]. In the early AD stages, DAM can be involved in preventing Aβ plaque-induced neuronal damage by inhibiting the spread of Aβ plaques through forming microglial barriers, a process that can be regulated by TREM2 [108,109,110,111]. Following DAM activation, which can occur independently of TREM2 and involve the activation of *APOE*4 and DAP12 genes as observed in TREM2-deficient mouse models, a TREM2-dependent program is triggered that can promote phagocytic pathways and lipid metabolism [111,112].

During the transition from homeostatic microglia to DAM, genes involved in lipid metabolism and phagocytosis are reported to be upregulated; however, without TREM2, the transition to Stage 2 DAM can be impaired. These findings reveal that TREM2 is essential for DAM development, and impairment in TREM2 signaling can result in significant disturbances in the degree and function of DAM activation. In neurodegenerative diseases, protein aggregation and neuronal damage trigger the activation of DAM through damage-associated molecular patterns (DAMPs), which can induce persistent inflammatory pathways and ROS production. Within microglia, ROS can be primarily generated by NADPH oxidase 2 (NOX2). Furthermore, NOX2 activation in DAM can be linked to DAMP signaling, inflammation, and the accumulation of amyloid-beta plaques [106].

## 5. TREM2 Mutations

**Figure 6:**
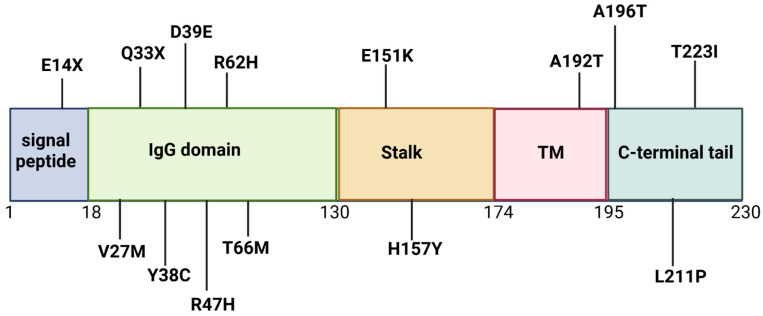
Examples of mutations in TREM2 protein across the different domains.

Due to the latest update of the Alzforum database (https://www.alzforum.org/mutations/trem2, accessed on 1 June 2025), 46 genetic mutations were identified in *TREM2* in patients diagnosed with various neurodegenerative diseases (Figure 6; Table 1). *TREM2* mutations involved in neurodegenerative diseases can be diverse, including single-amino-acid substitutions, frameshift mutations, nonsense mutations, and splice site-affecting variants [12]. While the majority of pathogenic *TREM2* mutations have been reported within the coding region, disease risk mutations (c.−5030G > C or c.−2986T > C) were also observed in the 3′UTR and in the upstream region of the transcription start site [61,107].

The first identified *TREM2* variants linked to neurodegenerative diseases were W78X and W44X, which cause premature protein truncation, leading to FTD or NHD pathogenicity. Furthermore, splice site variants were also identified, such as c.482 + 2T > C, which can abolish the donor site of *TREM2*, leading to exon skipping. Additionally, cell studies on *TREM2* Lys186Asn revealed that it could prevent the binding of TREM2 to DAP12, leading to abnormal inflammatory signaling. Representative genetic mutations reported in the *TREM2* gene include Arg47His, Arg62His, Thr66Met, and Gln33Ter, which are located in exon 2 of *TREM2* [18,113,114,115,116,117,118].

**Table 1:** Examples of mutations in *TREM2* gene and their clinical phenotypes.

Mutation	Gene Variant	Disease	Age of Onset	Imaging Data	Functional Data	Reference
Glu14Ter	40 G > T	NHD	NA	NA	Reduced sTREM2 in blood	[55]
Val27Met	79 G > A	AD	NA	NA	Not effect on TREM2 maturation, putative effects on ligand binding	[118]
Gln33Ter	97 C > T	AD, NHD, FTD	30 s–40 s	Bone cysts, cerebral atrophy: AD patient: typical AD pathology	Loss of TREM2 expression	[47,119]
Tyr38Cys	113 G > A	FTD	40 s	Cortical atrophy, white matter abnormalities	Disturbs ligand binding and TREN2 phagocytosis	[47]
Asp39Glu	140 G > A	AD, FTD	NA	NA	NA	[117]
Arg47His	117 C > G	AD, NHD, FTD, ALS	50 s	Severe gray-matter loss, lower microglial coverage of plaques	Elevated CSF-Tau, reduced ligand binding and microglial activation	[11,12]
Arg62His	185 C > T	AD	NA	Lower microglial coverage of plaques	Reduced ligand binding and microglial activation	[117,118]
Thr66Met	197 C > T	FTD	30 s	Frontal lobe atrophy, ventricular enlargement	Reduced cell surface expression of TREM2, impaired microglial activation	[47]
Glu151Lys	451 G > A	AD	NA	NA	Reduced normal TREM2 expression	[12,119]
His157Tyr	469 C > T	AD, FTD	NA	NA	Increased soluble TREM2 shedding, reduced phagocytosis	[115,116]
Ala192Thr	574 G > A	AD, FTD	50 s	hypometabolism in bilateral anterior temporal areas	Reduced cell surface expression of TREM2	[114,115]
Ala196Thr	586 G > A	AD	NA	NA	Probable reduced TREM2 cell surface expression	[111]
Leu211Pro	632 T > C	AD, FTD	NA	NA	Lower TREM2 CSF levels	[117]
Thr223Ile	668 C > T	AD, FTD	NA	NA	Slight changes in TREM2 maturation	[114]

### 5.1. TREM2 Arg47His

The *TREM2* Arg47His (rs759322628) mutation was verified as a strong genetic risk factor associated with AD, which could affect the function of TREM2 protein and could impact neurodegenerative diseases [11,12]. A recent study announced that the *TREM2* Arg47His mutation increased the risk for late-onset AD onset [119]. The *TREM2* Arg47His mutation was first reported by Jiang et al. (2013) in patients [119], diagnosed with frontotemporal dementia (FTD) and late-onset AD (LOAD). This study suggested that Arg47His could increase the risk for AD onset by two- to four-fold [119]. These findings revealed that *TREM2* Arg47His could be the second highest AD genetic risk factor following ApoE4 [11,12]. These findings also showed that additional rare mutations of *TREM2* could also play an important role in AD pathology. Cell studies revealed that *TREM2* Arg47His mutation reduced the TREM2 signaling after stimulation by ligands [103,120]. The mutation was found to decrease the TREM2 binding to phosphatidylserine (PS). TREM2-PS binding was verified to play a crucial role in activating the TREM2 signaling [121,122,123]. Furthermore, the Arg47His variant is extracellular region of TREM2 protein, leading to alterations in the glycosylation status, and increased the risk for developing AD [124]. Glycosylation status changes were verified to alter the ligand binding, functions of receptor, and TREM2 proteolysis, leading to neurodegeneration [124].

While the *TREM2* Arg47His mutation was found to be largely associated with an increased risk for AD, its role in tauopathies could present a more complex picture, since some studies suggested that the mutation may have a neuroprotective effect [122]. The PS19 mice expressing human *TREM2* Arg47His (PS19-T2R47H) showed significant attenuation in brain atrophy and lower degree of synapse loss compared to PS19 mice expressing the common variant of human *TREM2* (PS19-T2CV). These mouse models with *TREM2* Arg47His exhibited reduced microgliosis in different brain areas, such as hippocampus and piriform cortex compared to those without Arg47His. These neuroprotective effects may be related to the reduced degree of microglial phagocytosis by synapses through the reduced accumulation of C1q [122]. Also, in the later stages of AD, with advanced levels of Tau pathologies, or in case of pure Tau disease, the *TREM2* Arg47His mutation was suggested to slow down damage of neurons through reduced synaptic phagocytosis by the microglia [35]. Attenuation of TREM2 signaling reduced the effectiveness of microglial response to Tau pathology. Although partial or normal TREM2 activity was suggested to contribute to Tau disease or Tau-mediated damage, the full loss of TREM2 function could alleviate the Tau-mediated neuronal damage [125,126,127]. The *TREM2* Arg47His mutation reduced Tau-induced inflammation and the expression of pro-inflammatory factors and prevented microglial synaptic phagocytosis [122]. Attenuation of TREM2 signaling reduced the effectiveness of microglial response to Tau pathology. Although partial or normal TREM2 activity was suggested to contribute to Tau disease or Tau-mediated damage, the full loss of TREM2 function could alleviate the Tau-mediated neuronal damage [125,126,127]. The dual role of *TREM2* Arg47His highlights that the impact of microglial function in neurodegeneration could be highly context-dependent. In the stages of AD, where amyloid pathology dominates, the *TREM2* Arg47His should compromise the microglial ability to clear Aβ and prevent Aβ-induced Tau spreading. However, in later AD stages or in tauopathies, the partial loss of TREM2 function may be protective by reducing excessive microglial activation and aberrant synaptic phagocytosis. This complex interplay could reflect the importance of understanding the disease stage and the specific pathological drivers when considering TREM2 as a therapeutic target for AD [119,120,121,122,123,124,125,126,127].

The other issue with *TREM2* Arg47His is the difference between mouse and human models [128]. Unlike *TREM2* gene in humans, the Arg47His mutation in the mouse gene resulted in abnormal *TREM2* splicing, since the mRNA contained the codon for premature termination [38]. The mouse models with *TREM2* Arg47His showed abnormal mRNAs, which were rapidly cleared by the mRNA surveillance system, leading to reduced *TREM2* mRNA and protein levels. In humans, *TREM2* Arg47His resulted in direct impairment of microglia-related pathways [129]. These findings represented that it should be crucial to discover the differences between mouse models and human cell models in AD research and in case of applying data from mouse models to human diseases [130].

**Figure 7:**
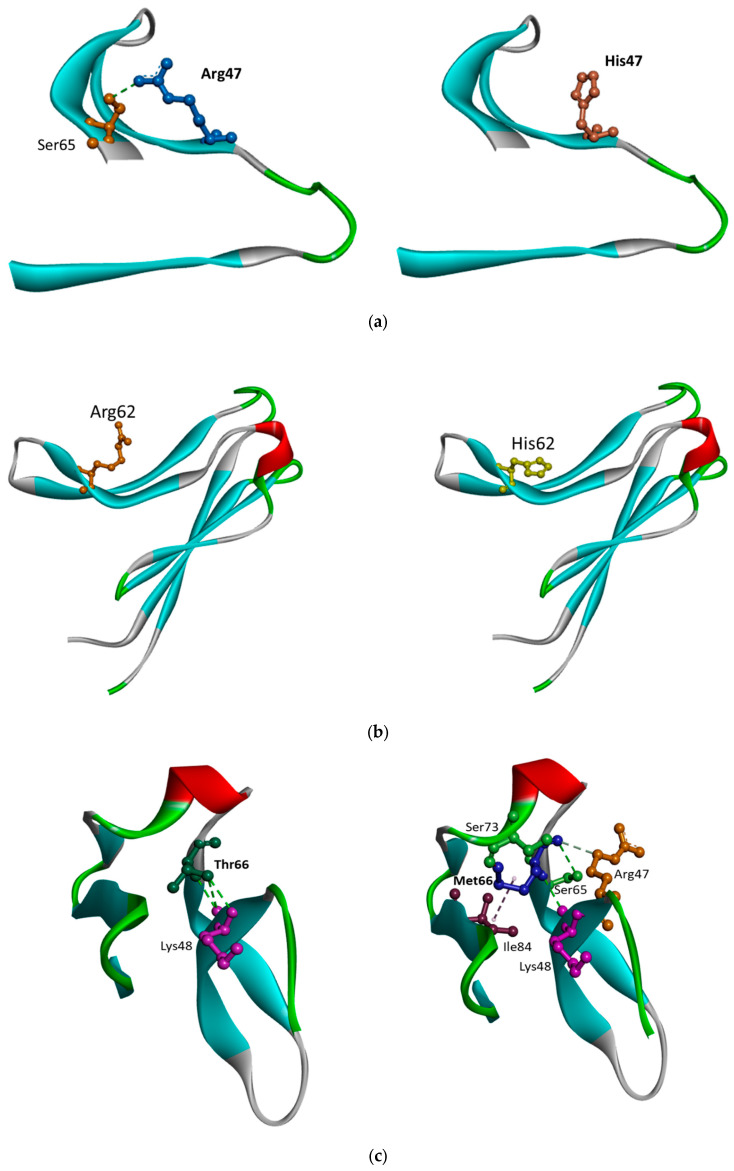

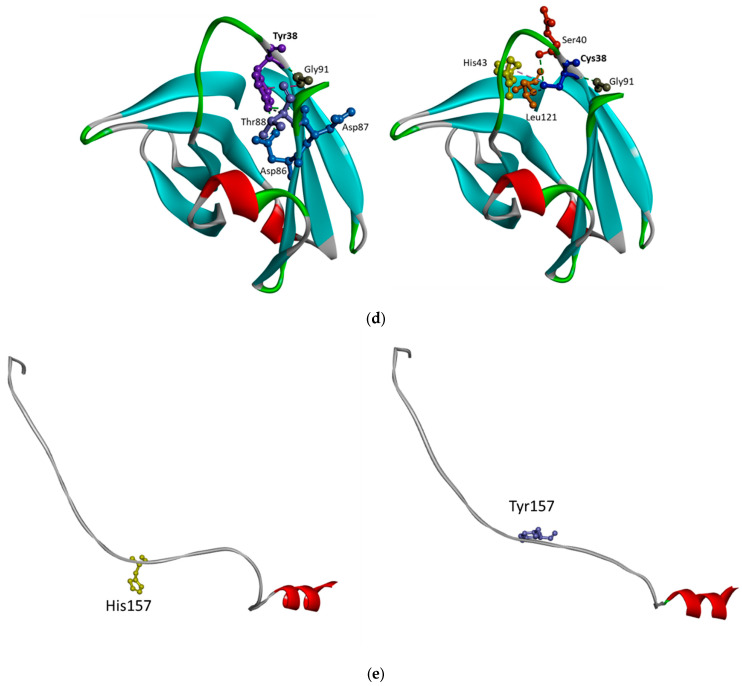
(**a**) Structure prediction on *TREM2* Arg47 vs. His47. (**b**) Structure prediction on *TREM2* R62 vs. H62. (**c**) Structure prediction on *TREM2* T66 vs. M66. (**d**) Structure prediction on *TREM2* Y38 vs. C38. (**e**) Structure prediction on *TREM2* H157 vs. Y157.

Structure predictions by AlphaFold Colab (https://colab.research.google.com/github/sokrypton/ColabFold/blob/main/AlphaFold2.ipynb, accessed on 1 June 2025) revealed that mutation may result in local changes in TREM2 IgG domain. *TREM2* Arg47 forms a hydrogen bond with Ser65. However, this hydrogen bond may be lost in the case of His47 (Figure 7a). Loss of this hydrogen bond can result in increased local flexibility or destabilization in that loop or β-sheet region. Arginine has a positively charged side chain, while histidine had a side chain with an imidazole ring. The mutation could potentially alter the intermolecular interactions too.

### 5.2. TREM2 Arg62His

The *TREM2* Arg62His (rs143332484) variant was first reported in a European American cohort and was revealed to be a strong Alzheimer’s disease (AD) risk factor. The variant was verified to impact AD risk in heterozygous form [114]. Even though the Arg62His variant was found to increase AD risk by 40–70%, the structural effect on the TREM2 protein was found to be weaker in Arg62His compared to the Arg47His mutation [108,113,114]. Both Arg47His and Arg62His affected TREM2 protein stability and its ligand binding [19,131]. Also, Arg62His altered the charge and size of the TREM2 surface, but caused less impairment in intramolecular interactions, including hydrogen bonds, compared to Arg47His [131]. Even though Arg62His may not result in as significant a change in protein structure or cell surface expression as Arg47His, it may impact TREM2 protein stability [132,133].

*TREM2* Arg62His may prevent the interaction between TREM2 and APOE, and it could affect the signaling pathway associated with microglial response regulation. By inhibiting TREM2-APOE interaction, *TREM2* Arg62His was associated with reduced intracellular absorption of the TREM2 protein [7]. In cell models with the *TREM2* Arg62His variant, the microglia failed to be localized properly around the amyloid plaques, leading to increased expression of TMEM119. Furthermore, the Arg62His mutation impaired the TREM2-mediated signaling of APOE, leading to the inhibition of microglial phagocytosis and reduced inflammatory response. Consequently, Arg62His may decrease the amyloid clearance by microglia and neuronal repair mechanisms, thereby contributing to neurodegeneration and AD progression [134].

*TREM2* Arg62His was suggested to have a relatively mild effect on the regulation of microglial ligand binding, including lipid ligands [117]. The reason could be that Arg62His may affect a specific TREM2 site, which could be responsible for the TREM2 interaction with APOE [135,136]. Since Arg62His may not strongly affect lipid ligand binding in general, the microglia may partially maintain the basic lipid metabolism and inflammatory response functions [103]. TREM2 was believed to have distinct binding sites: a specific extracellular domain for ligands, including APOE, and a general site that could recognize various lipids. The Arg62His mutation was suggested to alter the APOE binding site, leading to impaired TREM2 interaction with APOE, while leaving the other lipid-binding site intact. These selective binding differences may help to explain how Arg62His could affect microglial activity and offer valuable insights into AD pathogenesis [17,107,137]. Structure predictions revealed that Arg62His did not show significant changes in the intramolecular interactions and protein structure. No intramolecular interactions were predicted in case of R62 or H62. However, the arginine is highly positively charged, while in the case of histidine, there is more neural residue, which may result in stress inside the beta sheet dynamics (Figure 7b).

### 5.3. TREM2 Thr66Met and Tyr38Cys

Both Thr66Met and Tyr38Cys of *TREM2* were found to be associated with neurodegenerative diseases of NHD and FTD. The T66M mutation was verified by X-ray crystallography to result in misfolding of TREM2 protein within the Ig-like domain, leading to protein aggregation [17,138,139,140,141]. It was predicted that Thr66Met may destabilize the normal TREM2 protein structure, leading to stronger hydrophobic interactions, leading to higher tendency of protein aggregation. Indeed, unlike wild-type TREM2, *TREM2* with Thr66Met mainly existed in the aggregated form. This shows that this mutation reduced TREM2 structure stability and induced aggregation [139,140,141]. Thr66Met could disrupt the proper folding of the TREM2 protein, which could result in TREM2 protein accumulation in the endoplasmic reticulum (ER), and in preventing the secretion of sTREM2 by impairing its transport to the cell surface [17,142,143]. This accumulation could induce ER stress, which could contribute to microglial dysfunction, reduced cerebral perfusion, and impaired glucose metabolism. Because TREM2 was suggested to play a crucial role in maintaining brain homeostasis, these Thr66Met-induced disturbances hinder its function, potentially compromising microglial viability and activity [144,145]. Consequently, ER stress may cause by Thr66Met negatively, which could impact microglial survival and function [146].

Similar to Thr66Met, *TREM2* Tyr38Cys was associated with NHD and FTD, and it could also contribute to decreased TREM2 protein expression. *TREM2* Tyr38Cys also affected the structural stability of Ig-like domain in TREM2 protein, leading to abnormal folding process. Molecular modeling studies revealed that *TREM2* Tyr38Cys mutation may result in higher flexibility of TREM2 protein. The structural changes could impair the folding of Ig-like domain core region and reduce the stability of the entire TREM2 protein [146,147,148]. Furthermore, like Thr66Met, the Tyr38Cys mutation prevented the normal folding of TREM2 in the ER [147]. It was verified that migration of TREM2 protein with Tyr38Cys the ER was impaired, and it contributed to significantly lower cell surface expression of TREM2 [148]. Additionally, *TREM2* Tyr38Cys was found to cause bone mass loss, which could lead to NHD phenotypes that cause cognitive impairment and skeletal deterioration [146]. Structure predication on Thr66Met (Figure 7c) revealed significant changes in intramolecular interactions. *TREM2* with T66 formed three conventional H-bonds with K48 (length 2.92 A, 3.40 A, and 3 A). In the case of M66, only one H-bond remained with K48 (2.98 A). In the case of M66, an unfavorable bump was formed with S73 (1.91 A). M66 also formed a conventional hydrogen bond with S65 (3.27 A), and a carbon hydrogen bond with Arg47 (3.33 A). In the case of TREM2 Tyr38Cys (Figure 7d), a conventional hydrogen bond (length 2.66 A) and an amide stacked interaction (length: 3.59 A) was formed with Gly91. Y38 also formed a conventional H-bond with Asp86 (length 2.54 A) and with Asp387 (length 3.39 A), and a carbon hydrogen bond with Thr88 (length 3.32 A). In the case of Cys38, two conventional hydrogen bonds could be seen, Ser40 (length 3.79 A) and Gly91 (2.77 A). Cys38 forms pi-Alkyl bonds with His43 (distance: 5 A), and an alkyl bond with Leu121 (Distance 4.36 A).

### 5.4. His157Tyr

The *TREM2* His157Tyr mutation presents a complex and contradictory profile in neurodegenerative disease research, evidenced by its association with accelerated TREM2 protein shedding and AD risk, yet simultaneously exhibiting potential beneficial effects on synaptic plasticity and reduced amyloid pathology, with varying impacts across different patient populations [149,150,151,152,153,154]. The *TREM2* His157Tyr mutation presents a complex and contradictory profile in neurodegenerative disease research, evidenced by its association with accelerated TREM2 protein shedding and AD risk, yet simultaneously exhibits potential beneficial effects on synaptic plasticity and reduced amyloid pathology, with varying impacts across different patient populations [149,150,151,152]. The *TREM2* His157Tyr variant is associated with an increased risk of Alzheimer’s disease, particularly in the Han Chinese population [115]. A meta-analysis from 2019 found significant association between *TREM2* His157Tyr and AD risk [136]. However, some studies, including a replication study in a Japanese population, failed to find a significant association between the *TREM2* His157Tyr variant and AD [151]. This variability suggested that genetic and environmental factors may interact with the His157Tyr mutation to influence disease development [151,152,153,154,155]. Furthermore, heterozygous *TREM2* His157Tyr was also reported in FTD patients, where carriers presented early onset behavioral changes and cognitive impairment. Atrophy in their brain was also higher in different areas such as frontal, temporal, parietal, precuneus, and basal ganglia [64].

His157Tyr has been reported in the TREM2 extracellular domain and could affect the cleavage site of the TREM2 protein. Studies on His157Tyr have demonstrated that TREM2 with this mutation could be cleaved more easily, resulting in a higher degree of soluble sTREM2 production and increased sTREM2 levels in cerebrospinal fluid (CSF) and blood [152]. These findings suggest that the *TREM2* His157Tyr variant increases the activity of metalloproteases (such as ADAM19) for cleavage, which results in a higher degree of sTREM2 production and increased levels of TREM2 C-terminal fragment (CTF) formation [152,153]. It may also be possible that the mutation could cause enhanced degradation of the TREM2 CTF [149]. These results reveal that His157Tyr acts as a stimulator for TREM2 shedding. However, *TREM2* His157Tyr may not impact microglial density and functions. The mutation seems to selectively impact specific functions such as synaptic plasticity. However, the mutation has been suggested to reduce amyloid pathology [152]. A previous study suggested that the mutation could impact AD onset through amyloid-independent mechanisms, including Tau-related mechanisms [149,150,151,152,153].

Additional studies have reported that His157Tyr may result in more serious neuropsychological damage in FTD patients, while failing to find an association between His157Tyr and AD in certain population groups, such as Japanese or Chinese populations. These findings indicate that *TREM2* His157Tyr may interact with a variety of other genetic or environmental factors to influence disease development [149,150,151,152,153,154].

Structure prediction (Figure 7e) revealed that *TREM2* His157Tyr may not result in significance changes in the STALK region. However, the histidine is less hydrophobic, compared to the tyrosine, which may disturb this area.

### 5.5. TREM2 Gln33Ter

*TREM2* Gln33Ter is a mutation that results in a premature stop codon, leading to haploinsufficiency. This mutation was reported in both AD and NHD patients [155,156]. Gln33Ter was suggested increase the risk of LOAD in heterozygous patients and could be causative factor of EOAD in homozygous form [11,13]. The homozygous *TREM2* Gln33Ter was reported in two Italian sisters with NHD [156,157]. The Gln33Ter variant was also reported in patients, diagnosed with autosomal recessive behavioral variant frontotemporal dementia (bvFTD). However, these patients did not have any bone involvement, which was confirmed through radiology. This study suggested that Gln33Ter may be involved in autosomal recessive bvFTD-like syndrome without any bone pathology, which is the typical hallmark of NHD [49].

The Gln33Ter was verified to cause the loss of *TREM2* function [139]. The Gln33Ter mutation may result in microglial dysfunction, since cell studies revealed reduced that mutation carrier HeLa cells failed to express *TREM2*. However, sTREM2 was present in the plasma from homozygous carriers, which was lower, compared to those with normal TREM2, suggesting that there may be some kind of translation, even though the TREM2 was truncated [142,157].

## 6. TREM2 Variations and Therapeutic Targeting

TREM2 has been verified to bind to several types of ligands, such as phospholipids, lipoproteins, or apoptotic cells [6]. Pharmacologically, directly targeting the TREM2 receptor is considered likely to reduce both systemic effectiveness and specificity. Instead, activating the downstream signaling pathways of TREM2 is believed to offer improved systemic effects with greater specificity [98,100,127]. After TREM2 and its ligand interact, the tyrosine protein kinase SYK and phosphatidylinositol 3-kinase (PI3K) pathways are activated through the intracellular DAP12 and DAP10 adapter proteins. This results in the activation of different immune mechanisms, including microglial proliferation, phagocytosis, and the control of inflammatory response through intracellular signaling. TREM2 activation can trigger DAP12, which can induce microglial functions through PI3K or ERK downstream signaling pathways [98,100]. These pathways are crucial in promoting survival, proliferation, migration, and microglial phagocytosis, as well as in inhibiting the inflammatory response (Figure 4) [98,100]. Treatment strategies that induce the TREM2 downstream signaling pathway may have fewer side effects compared to strategies that target the TREM2 receptor directly. This is because the TREM2 signaling pathway may impact different cellular functions, selectively modulating specific molecules and resulting in minimized unnecessary side effects on the immune system [98,148].

Currently, research is focused on creating antibodies that can activate TREM2’s downstream signaling pathways. These antibodies have the benefit of targeting a specific site on TREM2 without entering the cells, thereby minimizing the risk of unpredictable side effects [98]. The most advanced TREM2-related strategy involves using a monoclonal antibody (mAb) that can bind to TREM2 selectively, leading to its activation without entering the cell. By targeting a specific region of TREM2, these antibodies may reduce the likelihood of unpredictable side effects compared to small molecule treatments, minimizing non-specific interactions with other receptors or cells. Furthermore, these mAbs (including AL002, Ab-T1, or CGX101) can enhance microglial phagocytosis, helping to clear waste products such as brain amyloid plaques [11,158,159,160,161,162,163,164]. Techniques aimed at targeting the extracellular domain of TREM2 are used to discover antibodies that can activate TREM2 signaling. These antibodies likely promote TREM2 signal transmission by inducing cross-linking [162,163]. Some studies, however, indicate that overactivation of TREM2 by activating antibodies may worsen existing amyloid pathology and amplify neuroinflammatory pathways. As a result, treatment approaches must be customized according to the disease stage and individual patient characteristics. Continued research is crucial to fully understand the specific functions and mechanisms of TREM2 [161,162,163,164,165,166,167,168]. Besides the antibodies, small molecule inhibitors of TREM2 were suggested to play a role in the anti-inflammatory effects and in the prevention of amyloid aggregation in the case of TREM2 dysfunctions [11,158,159,160,161,162,163,164].

Since the majority of TREM2 variants linked to Alzheimer’s disease (AD) are suggested to result in disease mechanisms through loss-of-function mechanisms, enhancing TREM2 expression—even in patients without any TREM2 mutations—has been proposed as an alternative therapeutic approach. This increase could be accomplished by using activating antibodies directed at TREM2 or by introducing TREM2 into myeloid cells externally and then administering them in vivo. Experiments are ongoing on gene therapy or cell therapy, which were suggested to restore the proper TREM2 and microglial functions [169,170,171]. Even though targeting TREM2 may be promising in the case of neurodegeneration, conflicting results were found in terms of whether TREM2 expression could be beneficial or harmful. Further studies are needed on the neurodegenerative and inflammatory mechanisms, which could be associated with TREM2 and sTREM2. Timing of treatment should be also a crucial, since the currently available therapeutic candidates may be more effective in the earlier disease stages. For example, in the early disease stages, TREM2 may be more effective in amyloid clearance. Genetic heterogeneity may also be a great challenge in TREM2 targeting therapies. Besides Arg47His or Arg62His, the role of other rare *TREM2* variants should also not be overlooked, since rare TREM2 variants may also be involved in neurodegeneration. Finding and developing precise biomarkers for microglial activation and for monitoring the real-time TREM2 singling may also be essential for therapeutic analysis and dose adjustment. Gene therapies for *TREM2* may be useful, but off-target effects should be avoided. Another challenge could be that stimulation of TREM2 may result in overactivation of microglia, leading to neurodegeneration. These challenges suggest that further research on TREM2 mutations, TREM2-related biomarkers, and optimizing the timing and dosing are essential [169,170,171,172].

Ongoing research is investigating the therapeutic potential of targeting *TREM2* (Table 2). VG-3927 is a selective, brain-penetrant, oral small molecule TREM2 agonist currently under development for Alzheimer’s disease (AD). It was designed to enhance protective microglial responses to aggregated amyloid and tau without increasing inflammation. VG-3927 could maximize receptor activation and microglial function because it does not bind to sTREM2. It could act as a molecular glue that potentiates the TREM2 signaling response to natural damage ligands. Preclinical data show that it enhances microglial amyloid and Tau uptake. VG-3927 has successfully completed Phase 1 clinical trials, since it was associated with reduced sTREM2 CSF levels, and it will be advanced into a Phase 2 trial, which Vigil Neuroscience plans to initiate in the third quarter of 2025. The primary limitation VG-3927 could be the complex and sometimes contradictory understanding of TREM2 function throughout AD progression. There is a concern that microglia expressing the most TREM2 might become desensitized to TREM2 stimulation, which could reduce the effectiveness of VG-3927 in some individuals. Additionally, further studies should be needed how changes in sTREM2 influence microglial activation, amyloid-beta plaque clearance, and clinical AD progression [163,164].

AL002 is a humanized monoclonal IgG1 antibody that activates the TREM2 signaling pathway to increase the ability of microglia to clear pathology and protect neurons. It binds to the stalk region of TREM2, which activates signaling but also promotes partial internalization of the receptor, and it could inhibit the shedding of soluble TREM2. AL002 completed a Phase 1 study (INVOKE) in healthy volunteers, which showed it was generally well tolerated and demonstrated proof of target engagement and mechanism. Based on these results, AL002 progressed to the INVOKE-2 Phase 2 study in patients with early AD. The INVOKE-2 trial failed to meet its primary endpoint of slowing decline and also did not show promising improvements in CSF biomarkers, or amyloid-PET. The long-term extension study of AL002 has been halted, and its future status remains unknown. Further studies are needed on optimizing AL002 dosage, treatment timing, and the possibility of combining it with other therapies [165,166].

Several drug candidates are available, currently in the preclinical phase. CGX101 is an IgG4 monoclonal antibody that targets the extracellular domain of TREM2. This antibody was designed to target both membrane-bound and soluble forms of TREM2. It could target sTREM2; CGX101 could neutralize its pro-inflammatory effects and protect against the long-term chronic neuroinflammation. Studies on 5xFAD mice revealed that CGX101 could reduce the amyloid deposition and phospho-Tau burden. Furthermore, it may improve the cognitive functions in AD mice [161,167,168]. The Ab-T1 antibody is also a monoclonal antibody, which could target both membrane-bond and sTREM2 with high affinity. It could bind to the extracellular domain of TREM2, and activate the microglia though Syk and Akt pathways, leading to the uptake of oligomeric amyloid peptides and apoptotic neurons. Experiments on 5xFAD mice also revealed that Ab-T1 could also downregulate the pro-inflammatory cytokines and improve the cognitive functions. Furthermore, Ab-T1 was tested in CSF from human AD patients, and it reduced the sTREM2 levels in them [161]. 4D9 is a monoclonal antibody that targets TREM2 and it functions by stabilizing the full-length TREM2 protein on the cell surface, reducing its proteolytic shedding by α-secretase. It also activates phospho-SYK signaling pathways. This antibody binds to the stalk region epitope of TREM2, which is close to the cleavage site. It could activate the microglia and increase the amyloid clearance. In addition, 4D9 antibody could induce the mitochondrial functions and enhance the brain metabolism. Mouse models showed that 4D9 could enhance the TREM2 signaling. One of its limitations could be that it targets not only membrane bond TREM2, but also sTREM2 [169]. Ab18 is a tetravalent agonistic monoclonal antibody that aims to increase TREM2 activation, leading to increased amyloid clearance. Mouse experiments suggested that Ab18 may result in reduced amyloid and Tau levels. Furthermore, the synaptic markers were also increased, and the cognitive functions were also improved in the mice. One of the limitations of Ab18 could be that overstimulated TREM2 could result in microglial overactivation, which could result in neurodegeneration [170].

Besides antibodies, gene therapies and cell therapies may also show promise in the development of TREM2-related therapies [171,172,173]. PR-009 is an AAV-based gene therapy designed to increase TREM2 levels in patients and microglial functions through inducing TREM2-DAP12 interaction. One of the limitations could be the that TREM2 may act as a double-edged sword controlling the pro-inflammatory pathways [172]. Transplantation of Trem2+/+ circulation-derived myeloid cells can restore microglial function and replace mutant microglia. In AD mouse models, this cell therapy was associated with restored microglial functions and amyloid clearance. However, the issue with cell therapy could be that these myeloid cells may be associated with high mortality [173].

## 7. Conclusions

*TREM2* was verified as a risk factor for several neurodegenerative diseases, such as AD, FTD, NHD, and PD, suggesting a broader role in immune responses and maintaining nervous system homeostasis. However, the impact of *TREM2* mutation may not be straightforward, exhibiting complex and sometimes contradictory effects that necessitate a critical examination of current research [15,16]. Notably, mutations in *TREM2* could have diverse effects, such as impaired microglial function, reducing their ability to clear amyloid-beta and control inflammation, which can accelerate disease progression. Furthermore, they could enhance the neuroinflammatory process. However, besides increasing the degree of amyloid deposition, *TREM2* Arg47His was also found to be beneficial in the case of Tau pathology. Also, His157Tyr mutation was suggested to protect against amyloid pathology, but GWAS studies suggested that it could be a possible risk variant for AD. This duality reflected that TREM2 could maintain the immune response balance in the brain, where its effects could be both protective and pathological depending on the specific disease context or stage [118,119,120,121,122,123,124,125,126,127,128,129,130,131,132,133,134,135,136,137,138,139,140,141,142,143,144,145,146,147,148,149,150,151,152,153,154,155,156,157,158,159]. Investigating *TREM2* mutations is vital for understanding the pathological mechanisms underlying neurodegeneration and offers a foundation for developing novel therapeutic strategies [111,112,113,114,115,116,117,118,119,120,121,122,123,124,125,126,127,128,129,130,131,132,133,134,135,136,137,138,139,140,141,142,143,144,145,146,147,148,149,150,151,152,153,154,155,156,157,158,159,160,161,162,163,164,165,166,167,168,169,170,171,172,173]. Therapeutic approaches targeting TREM2—including antibodies or gene therapy [111,112,113,114,115,116,117,118,119,120,121,122,123,124,125,126,127,128,129,130,131,132,133,134,135,136,137,138,139,140,141,142,143,144,145,146,147,148,149,150,151,152,153,154,155,156,157,158,159,160,161,162,163,164,165,166,167,168,169,170,171,172,173]—should be promising approaches in AD, but they may require careful optimization based on disease stage, mutation type, and microglial state. After the unsuccessful clinical trial of INVOKE-2, there could be the question of whether TREM2 could be still a worthy target. A recent study suggested that it could be, but also it may not be. Targeting TREM2 may be still a promising area of drug development. However, further research may be needed on TREM2 therapies to find out the optimal dosage and timing of therapy. These results highlight that the microglial signaling may be very complex and a more refined and personalized approach should be essential in case of therapy. TREM2 function could be modulated by multiple factors, including mutations of disease stage, or biomarkers. Mutations in TREM2 may alter the gene and protein expression and sTREM2 levels, suggesting that identifying the genetic disease risk factors should be essential for drug development. Furthermore, using fluid and imaging biomarkers should also be important for monitoring the treatment outcome. TREM2 pathway engagement and monitoring therapeutic outcomes. sTREM2, a marker of microglial activation, has emerged as a potential dynamic indicator of treatment response [165,166]. Ultimately, a comprehensive understanding of the TREM2–microglia axis will be crucial for designing safe and effective interventions that can truly modify disease progression and improve outcomes for individuals affected by these devastating conditions [16,158,159,160,161,162,163,164,165,166,167,168,169,170,171,172,173,174].

## Figures and Tables

**Table 2:** Potential therapeutic candidates, targeting TREM2.

Name of Candidate	Type	Preclinical Development Results	Results in Clinical Trials	References
VG-3927	Small Molecule Agonist	iPSC: increased anti-inflammatory activation hTREM2-5xFAD mice: reduced amyloid aggregates	Phase 1: reduced sTREM2 levels (50%) May be safe, further research needed, since there may be potential side effects	[163,164]
AL002-humanized IgG antibody	Monoclonal antibody	Increases microglial activation, proliferation and survival	Phase 2: failed to slow down AD despite microglial activation	[165,166]
CGX101: IgG4 antibody	Monoclonal antibody	In vitro and 5xFAD mice: reduced amyloid burden and cognitive decline Reduced p-Tau in mice	Currently in preclinical development phase	[161,168]
Ab-T1	monoclonal antibody	Increased TREM2 expression, amyloid and apoptotic neuron uptake, reduced cognitive decline	Currently in preclinical development phase, but reduced sTREM2 levels in CSF from patients	[161]
4D9	Monoclonal antibody	Mouse models: protecting the microglia, enhanced TREM2 signaling, improved brain metabolism	Currently in preclinical development phase	[169]
Ab18	Monoclonal antibody	Rodent models: increased amyloid clearance, improved synaptic marker intensity, reduced Tau phosphorylation	Currently in preclinical development phase	[170]
PR009	Gene therapy	Stimulated TREM2 expression, maintain microglial balance	Currently in preclinical development phase	[172]
Transplantation of Trem2+/+ circulation-derived myeloid cells	Cell/Gene therapy	5xFAD mice: restored microglial functions, DAM expression, reduced plaque load	Currently in preclinical development phase	[173]

## References

[1] Zgorzynska E. (2024). TREM2 in Alzheimer’s disease: Structure, function, therapeutic prospects, and activation challenges. Mol. Cell. Neurosci..

[2] Ulland T.K., Colonna M. (2018). TREM2—A key player in microglial biology and Alzheimer disease. Nat. Rev. Neurol..

[3] Hou J., Chen Y., Grajales-Reyes G., Colonna M. (2022). TREM2 dependent and independent functions of microglia in Alzheimer’s disease. Mol. Neurodegener..

[4] Wu K.-M., Zhang Y.-R., Huang Y.-Y., Dong Q., Tan L., Yu J.-T. (2021). The role of the immune system in Alzheimer’s disease. Ageing Res. Rev..

[5] Carmona S., Hardy J., Guerreiro R., Geschwind D.H., Paulson H.L., Klein C. (2018). Chapter 26—The genetic landscape of Alzheimer disease. Handbook of Clinical Neurology.

[6] Deczkowska A., Weiner A., Amit I. (2020). The physiology, pathology, and potential therapeutic applications of the TREM2 signaling pathway. Cell.

[7] Gratuze M., Leyns C.E., Holtzman D.M. (2018). New insights into the role of TREM2 in Alzheimer’s disease. Mol. Neurodegener..

[8] Li Y., Xu H., Wang H., Yang K., Luan J., Wang S. (2023). TREM2: Potential therapeutic targeting of microglia for Alzheimer’s disease. Biomed. Pharmacotherapy.

[9] Greven J.A., Alexander-Brett J.M., Brett T.J. (2024). Structural and functional analysis of TREM2 interactions with amyloid beta reveal molecular mechanisms that drive phagocytosis of oligomeric amyloid beta. Alzheimer’s Dement..

[10] Wang Y., Ulland T.K., Ulrich J.D., Song W., Tzaferis J.A., Hole J.T., Yuan P., Mahan T.E., Shi Y., Gilfillan S. (2016). TREM2-mediated early microglial response limits diffusion and toxicity of amyloid plaques. J. Exp. Med..

[11] Park J.-C., Han J.W., Lee W., Kim J., Lee S.E., Lee D., Choi H., Han J., Kang Y.J., Diep Y.N. (2024). Microglia Gravitate toward Amyloid Plaques Surrounded by Externalized *Phosphatidylserine* via TREM2. Adv. Sci..

[12] Ulrich J.D., Holtzman D.M. (2016). TREM2 function in Alzheimer’s disease and neurodegeneration. ACS Chem. Neurosci..

[13] Guerreiro R., Wojtas A., Bras J., Carrasquillo M., Rogaeva E., Majounie E., Cruchaga C., Sassi C., Kauwe J.S., Younkin S. (2013). TREM2 variants in Alzheimer’s disease. N. Engl. J. Med..

[14] Jonsson T., Stefansson H., Steinberg S., Jonsdottir I., Jonsson P.V., Snaedal J., Bjornsson S., Huttenlocher J., Levey A.I., Lah J.J. (2013). Variant of TREM2 associated with the risk of Alzheimer’s disease. N. Engl. J. Med..

[15] Hickman S.E., El Khoury J. (2014). TREM2 and the neuroimmunology of Alzheimer’s disease. Biochem. Pharmacol..

[16] Dean H.B., Roberson E.D., Song Y. (2019). Neurodegenerative Disease-Associated Variants in TREM2 Destabilize the Apical Ligand-Binding Region of the Immunoglobulin Domain. Front. Neurol.

[17] Olufunmilayo E.O., Holsinger R.D. (2022). Variant TREM2 signaling in Alzheimer’s disease. J. Mol. Biol..

[18] Bailey C.C., DeVaux L.B., Farzan M. (2015). The Triggering Receptor Expressed on Myeloid Cells 2 Binds Apolipoprotein E. J. Biol. Chem..

[19] Kober D.L., Brett T.J. (2017). TREM2-Ligand Interactions in Health and Disease. J. Mol. Biol..

[20] Shaw B.C., Snider H.C., Turner A.K., Zajac D.J., Simpson J.F., Estus S. (2022). An Alternatively Spliced TREM2 Isoform Lacking the Ligand Binding Domain is Expressed in Human Brain. J. Alzheimer’s Dis..

[21] Li R.-Y., Qin Q., Yang H.C., Wang Y.Y., Mi Y.X., Yin Y.S., Wang M., Yu C.J., Tang Y. (2022). TREM2 in the pathogenesis of AD: A lipid metabolism regulator and potential metabolic therapeutic target. Mol. Neurodegener..

[22] Zhao Y., Wu X., Li X., Jiang L.L., Gui X., Liu Y., Sun Y., Zhu B., Piña-Crespo J.C., Zhang M. (2018). TREM2 is a receptor for β-amyloid that mediates microglial function. Neuron.

[23] Poliani P.L., Wang Y., Fontana E., Robinette M.L., Yamanishi Y., Gilfillan S., Colonna M. (2015). TREM2 sustains microglial expansion during aging and response to demyelination. J. Clin. Investig..

[24] Magno L., Bunney T.D., Mead E., Svensson F., Bictash M.N. (2021). TREM2/PLCγ2 signalling in immune cells: Function, structural insight, and potential therapeutic modulation. Mol. Neurodegener..

[25] Cignarella F., Filipello F., Bollman B., Cantoni C., Locca A., Mikesell R., Manis M., Ibrahim A., Deng L., Benitez B.A. (2020). TREM2 activation on microglia promotes myelin debris clearance and remyelination in a model of multiple sclerosis. Acta Neuropathol..

[26] Yeh F.L., Hansen D.V., Sheng M. (2017). TREM2, microglia, and neurodegenerative diseases. Trends Mol. Med..

[27] Hardy J. (1997). The Alzheimer family of diseases: Many etiologies, one pathogenesis?. Proc. Natl. Acad. Sci. USA.

[28] Small G.W. (1998). The pathogenesis of Alzheimer’s disease. J. Clin. Psychiatry.

[29] Hu N., Tan M.S., Yu J.T., Sun L., Tan L., Wang Y.L., Jiang T., Tan L. (2013). Increased expression of TREM2 in peripheral blood of Alzheimer’s disease patients. J. Alzheimer’s Dis..

[30] Prokop S., Miller K.R., Labra S.R., Pitkin R.M., Hoxha K., Narasimhan S., Changolkar L., Rosenbloom A., Lee V.M., Trojanowski J.Q. (2019). Impact of TREM2 risk variants on brain region-specific immune activation and plaque microenvironment in Alzheimer’s disease patient brain samples. Acta Neuropathol..

[31] Forabosco P., Ramasamy A., Trabzuni D., Walker R., Smith C., Bras J., Levine A.P., Hardy J., Pocock J.M., Guerreiro R. (2013). Insights into TREM2 biology by network analysis of human brain gene expression data. Neurobiol. Aging.

[32] Heslegrave A., Heywood W., Paterson R., Magdalinou N., Svensson J., Johansson P., Öhrfelt A., Blennow K., Hardy J., Schott J. (2016). Increased cerebrospinal fluid soluble TREM2 concentration in Alzheimer’s disease. Mol. Neurodegener..

[33] Franzmeier N., Suárez-Calvet M., Frontzkowski L., Moore A., Hohman T.J., Morenas-Rodriguez E., Nuscher B., Shaw L., Trojanowski J.Q., Dichgans M. (2020). Higher CSF sTREM2 attenuates ApoE4-related risk for cognitive decline and neurodegeneration. Mol. Neurodegener..

[34] Basha Sk C., Mekala J.R. (2024). Basic Science and Pathogenesis. Alzheimer’s Dement. J. Alzheimer’s Assoc..

[35] Lyu S., Lan Z., Li C. (2023). The triggering receptor expressed on myeloid cells 2–apolipoprotein E signaling pathway in diseases. Chin. Med. J..

[36] Wang Y.-C., Huang L.Y., Guo H.H., Liu M., Zhang Y.Y., Zhang Z.Q., Hao Q., Tan C.C., Tan L. (2024). Higher CSF sTREM2 attenuates APOE ε4-related risk for amyloid pathology in cognitively intact adults: The CABLE study. J. Neurochem..

[37] Lietzke E.E., Saeb D., Aldrich E.C., Bruce K.D., Sprenger K.G. (2025). Synergistic reduction in interfacial flexibility of TREM2R47H and ApoE4 may underlie AD pathology. Alzheimer’s Dement..

[38] Heneka M.T. (2023). ApoE4 makes microglia trem2bling. Neuron.

[39] Knapskog A.-B., Henjum K., Idland A.V., Eldholm R.S., Persson K., Saltvedt I., Watne L.O., Engedal K., Nilsson L.N.G. (2020). Cerebrospinal fluid sTREM2 in Alzheimer’s disease: Comparisons between clinical presentation and AT classification. Sci. Rep..

[40] Nabizadeh F., Seyedmirzaei H., Karami S. (2024). Neuroimaging biomarkers and CSF sTREM2 levels in Alzheimer’s disease: A longitudinal study. Sci. Rep..

[41] Crook H., Wahdan M., Tuil M.E., Livingston N.R., Raza S., Nowell J., Edison P. (2024). CSF sTREM2 is associated with neuroprotective microglial states early in Alzheimer’s disease and deleterious effects later in the disease trajectory. Alzheimer’s Dement..

[42] Španić Popovački E., Babić Leko M., Langer Horvat L., Brgić K., Vogrinc Ž., Boban M., Klepac N., Borovečki F., Šimić G. (2023). Soluble TREM2 concentrations in the cerebrospinal fluid correlate with the severity of neurofibrillary degeneration, cognitive impairment, and inflammasome activation in Alzheimer’s disease. Neurol. Int..

[43] Wang S., Chenghui C., Peng D. (2025). The various roles of TREM2 in cardiovascular disease. Front. Immunol..

[44] Li T., Lyu D., Liu F.-Q. (2022). Cerebrospinal Fluid sTREM2 in Alzheimer’s Disease Is Associated with Both Amyloid and Tau Pathologies but not with Cognitive Status. J. Alzheimer’s Dis..

[45] Park S.H., Lee E.H., Kim H.J., Jo S., Lee S., Seo S.W., Park H.H., Koh S.H., Lee J.H. (2021). The relationship of soluble TREM2 to other biomarkers of sporadic Alzheimer’s disease. Sci. Rep..

[46] Paloneva J., Manninen T., Christman G., Hovanes K., Mandelin J., Adolfsson R., Bianchin M., Bird T., Miranda R., Salmaggi A. (2002). Mutations in two genes encoding different subunits of a receptor signaling complex result in an identical disease phenotype. Am. J. Hum. Genet..

[47] Guerreiro R.J., Lohmann E., Brás J.M., Gibbs J.R., Rohrer J.D., Gurunlian N., Dursun B., Bilgic B., Hanagasi H., Gurvit H. (2013). Using exome sequencing to reveal mutations in TREM2 presenting as a frontotemporal dementia–like syndrome without bone involvement. JAMA Neurol..

[48] Zhou Y., Tada M., Cai Z., Andhey P.S., Swain A., Miller K.R., Gilfillan S., Artyomov M.N., Takao M., Kakita A. (2023). Human early-onset dementia caused by DAP12 deficiency reveals a unique signature of dysregulated microglia. Nat. Immunol..

[49] Williamson J.C., Larner A.J. (2019). Behavioral Variant Frontotemporal Dementia-like Syndrome with Novel Heterozygous TREM2 Frameshift Mutation. Alzheimer Dis. Assoc. Disord..

[50] Paloneva J., Kestilä M., Wu J., Salminen A., Böhling T., Ruotsalainen V., Hakola P., Bakker A.B., Phillips J.H., Pekkarinen P. (2000). Loss-of-function mutations in TYROBP (DAP12) result in a presenile dementia with bone cysts. Nat. Genet..

[51] Klunemann H., Ridha B.H., Magy L., Wherrett J.R., Hemelsoet D.M., Keen R.W., De Bleecker J.L., Rossor M.N., Marienhagen J., Klein H.E. (2005). The genetic causes of basal ganglia calcification, dementia, and bone cysts: DAP12 and TREM2. Neurology.

[52] Xing J., Titus A.R., Humphrey M.B. (2015). The TREM2-DAP12 signaling pathway in Nasu-Hakola disease: A molecular genetics perspective. Res. Rep. Biochem..

[53] Tinkler S.M., Linder J.E., Williams D.M., Johnson N.W. (1981). Formation of osteoclasts from blood monocytes during 1 alpha-OH Vit D-stimulated bone resorption in mice. J. Anat..

[54] Cella M., Buonsanti C., Strader C., Kondo T., Salmaggi A., Colonna M. (2003). Impaired differentiation of osteoclasts in TREM-2–deficient individuals. J. Exp. Med..

[55] Paloneva J., Mandelin J., Kiialainen A., Bohling T., Prudlo J., Hakola P., Haltia M., Konttinen Y.T., Peltonen L., Mandelin J. (2003). DAP12/TREM2 deficiency results in impaired osteoclast differentiation and osteoporotic features. J. Exp. Med..

[56] Konishi H., Kiyama H. (2018). Microglial TREM2/DAP12 Signaling: A Double-Edged Sword in Neural Diseases. Front. Cell Neurosci..

[57] Sasaki A., Kakita A., Yoshida K., Konno T., Ikeuchi T., Hayashi S., Matsuo H., Shioda K. (2015). Variable expression of microglial DAP12 and TREM2 genes in Nasu-Hakola disease. Neurogenetics.

[58] Mecca C., Giambanco I., Donato R., Arcuri C. (2018). Microglia and Aging: The Role of the TREM2-DAP12 and CX3CL1-CX3CR1 Axes. Int. J. Mol. Sci..

[59] Cady J., Koval E.D., Benitez B.A., Zaidman C., Jockel-Balsarotti J., Allred P., Baloh R.H., Ravits J., Simpson E., Appel S.H. (2014). TREM2 variant p. R47H as a risk factor for sporadic amyotrophic lateral sclerosis. JAMA Neurol..

[60] Borroni B., Ferrari F., Galimberti D., Nacmias B., Barone C., Bagnoli S., Fenoglio C., Piaceri I., Archetti S., Bonvicini C. (2014). Heterozygous TREM2 mutations in frontotemporal dementia. Neurobiol. Aging.

[61] Cuyvers E., Bettens K., Philtjens S., Van Langenhove T., Gijselinck I., van der Zee J., Engelborghs S., Vandenbulcke M., Van Dongen J., Geerts N. (2014). Investigating the role of rare heterozygous TREM2 variants in Alzheimer’s disease and frontotemporal dementia. Neurobiol. Aging.

[62] Rayaprolu S., Mullen B., Baker M., Lynch T., Finger E., Seeley W.W., Hatanpaa K.J., Lomen-Hoerth C., Kertesz A., Bigio E.H. (2013). TREM2 in neurodegeneration: Evidence for association of the p. R47H variant with frontotemporal dementia and Parkinson’s disease. Mol. Neurodegener..

[63] Thelen M., Razquin C., Hernández I., Gorostidi A., Sánchez-Valle R., Ortega-Cubero S., Wolfsgruber S., Drichel D., Fliessbach K., Duenkel T. (2014). Investigation of the role of rare TREM2 variants in frontotemporal dementia subtypes. Neurobiol. Aging.

[64] Ogonowski N., Santamaria-Garcia H., Baez S., Lopez A., Laserna A., Garcia-Cifuentes E., Ayala-Ramirez P., Zarante I., Suarez-Obando F., Reyes P. (2023). Frontotemporal dementia presentation in patients with heterozygous p.H157Y variant of TREM2. J. Med. Genet..

[65] Xie M., Liu Y.U., Zhao S., Zhang L., Bosco D.B., Pang Y.P., Zhong J., Sheth U., Martens Y.A., Zhao N. (2022). TREM2 interacts with TDP-43 and mediates microglial neuroprotection against TDP-43-related neurodegeneration. Nat. Neurosci..

[66] Xie M. (2022). The Role of Microglia TREM2 in Tdp-43 Related Neurodegeneration. Ph.D. Thesis.

[67] Mills W.A., Eyo U.B. (2023). TREMble before TREM2: The mighty microglial receptor conferring neuroprotective properties in TDP-43 mediated neurodegeneration. Neurosci. Bull..

[68] Wang X., Hu Y., Xu R. (2024). The pathogenic mechanism of TAR DNA-binding protein 43 (TDP-43) in amyotrophic lateral sclerosis. Neural Regen. Res..

[69] Greven J.A., Wydra J.R., Greer R.A., Zhi C., Price D.A., Svoboda J.D., Camitta C.L.M., Washington M., Leung D.W., Song Y. (2025). Biophysical mapping of TREM2-ligand interactions reveals shared surfaces for engagement of multiple Alzheimer’s disease ligands. Mol. Neurodegener..

[70] Seddighi S., Qi Y.A., Brown A.L., Wilkins O.G., Bereda C., Belair C., Zhang Y.J., Prudencio M., Keuss M.J., Khandeshi A. (2024). Mis-spliced transcripts generate de novo proteins in TDP-43–related ALS/FTD. Sci. Transl. Med..

[71] Chhangani D., Rincon-Limas D.E. (2022). TDP-35, a truncated fragment of TDP-43, induces dose-dependent toxicity and apoptosis in flies. Neural Regen. Res..

[72] Li X.X., Zhang F. (2021). Targeting TREM2 for Parkinson’s Disease: Where to Go?. Front. Immunol.

[73] Huang P., Zhang Z., Zhang P., Feng J., Xie J., Zheng Y., Liang X., Zhu B., Chen Z., Feng S. (2024). TREM2 Deficiency Aggravates NLRP3 Inflammasome Activation and Pyroptosis in MPTP-Induced Parkinson’s Disease Mice and LPS-Induced BV2 Cells. Mol. Neurobiol..

[74] Huang W., Huang W., Lv Q., Xiao Y., Zhong Z., Hu B., Yan S., Yan Y., Zhang J., Shi T. (2021). Triggering receptor expressed on myeloid cells 2 protects dopaminergic neurons by promoting autophagy in the inflammatory pathogenesis of Parkinson’s disease. Front. Neurosci..

[75] Liu Z., Ning J., Zheng X., Meng J., Han L., Zheng H., Zhong L., Chen X.F., Zhang X., Luo H. (2020). TMEM59 interacts with TREM2 and modulates TREM2-dependent microglial activities. Cell Death Dis..

[76] Lv Q., Zhong Z., Hu B., Yan S., Yan Y., Zhang J., Shi T., Jiang L., Li W., Huang W. (2021). MicroRNA-3473b regulates the expression of TREM2/ULK1 and inhibits autophagy in inflammatory pathogenesis of Parkinson disease. J. Neurochem..

[77] Jay T.R., von Saucken V.E., Landreth G.E. (2017). TREM2 in Neurodegenerative Diseases. Mol. Neurodegener..

[78] Guo Y., Wei X., Yan H., Qin Y., Yan S., Liu J., Zhao Y., Jiang F., Lou H. (2019). TREM2 deficiency aggravates α-synuclein-induced neurodegeneration and neuroinflammation in Parkinson’s disease models. FASEB J..

[79] Dela Cruz H.L., Dela Cruz E.L., Zurhellen C.J., York H.T., Baun J.A., Dela Cruz J.L., Dela Cruz J.S. (2023). New insights underlying the early events of dopaminergic dysfunction in Parkinson’s Disease. bioRxiv.

[80] Eo H., Kim S., Jung U.J., Kim S.R. (2024). Alpha-Synuclein and Microglia in Parkinson’s Disease: From Pathogenesis to Therapeutic Prospects. J. Clin. Med..

[81] Deyell J.S., Sriparna M., Ying M., Mao X. (2023). The Interplay between α-Synuclein and Microglia in α-Synucleinopathies. Int. J. Mol. Sci..

[82] Tsunemi T., Krainc D. (2014). Zn^2+^ dyshomeostasis caused by loss of ATP13A2/PARK9 leads to lysosomal dysfunction and alpha-synuclein accumulation. Hum. Mol. Genet..

[83] Yin S., Chi X., Wan F., Li Y., Zhou Q., Kou L., Sun Y., Wu J., Zou W., Wang Y. (2024). TREM2 signaling in Parkinson’s disease: Regulation of microglial function and α-synuclein pathology. Int. Immunopharmacol..

[84] Shan H.-M., Zang M., Zhang Q., Shi R.B., Shi X.J., Mamtilahun M., Liu C., Luo L.L., Tian X., Zhang Z. (2020). Farnesoid X receptor knockout protects brain against ischemic injury through reducing neuronal apoptosis in mice. J. Neuroinflamm..

[85] Singaraja R.R. (2013). TREM2: A new risk factor for Alzheimer’s disease. Clin. Genet..

[86] Paradowska-Gorycka A., Jurkowska M. (2013). Structure, expression pattern and biological activity of molecular complex TREM-2/DAP12. Hum. Immunol..

[87] Bouchon A., Hernández-Munain C., Cella M., Colonna M. (2001). A DAP12-mediated pathway regulates expression of CC chemokine receptor 7 and maturation of human dendritic cells. J. Exp. Med..

[88] Zhong L., Chen X.F., Zhang Z.L., Wang Z., Shi X.Z., Xu K., Zhang Y.W., Xu H., Bu G. (2015). DAP12 Stabilizes the C-terminal Fragment of the Triggering Receptor Expressed on Myeloid Cells-2 (TREM2) and Protects against LPS-induced Pro-inflammatory Response. J. Biol. Chem..

[89] Ewers M., Franzmeier N., Suárez-Calvet M., Morenas-Rodriguez E., Caballero M.A.A., Kleinberger G., Piccio L., Cruchaga C., Deming Y., Dichgans M. (2019). Increased soluble TREM2 in cerebrospinal fluid is associated with reduced cognitive and clinical decline in Alzheimer’s disease. Sci. Transl. Med..

[90] Glass C.K., Saijo K., Winner B., Marchetto M.C., Gage F.H. (2010). Mechanisms underlying inflammation in neurodegeneration. Cell.

[91] Chen Y., Yu Y. (2023). Tau and neuroinflammation in Alzheimer’s disease: Interplay mechanisms and clinical translation. J. Neuroinflammation.

[92] Zhang J., Zhang Y., Wang J., Xia Y., Zhang J., Chen L. (2024). Recent advances in Alzheimer’s disease: Mechanisms, clinical trials and new drug development strategies. Signal Transduct. Target. Ther..

[93] Amos P.J., Fung S., Case A., Kifelew J., Osnis L., Smith C.L., Green K., Naydenov A., Aloi M., Hubbard J.J. (2017). Modulation of hematopoietic lineage specification impacts TREM2 expression in microglia-like cells derived from human stem cells. ASN Neuro.

[94] Wang M., Gao X., Zhao K., Chen H., Xu M., Wang K. (2019). Effect of TREM2 on release of inflammatory factor from LPS-stimulated microglia and its possible mechanism. Ann. Clin. Lab. Sci..

[95] Novoa C., Salazar P., Cisternas P., Gherardelli C., Vera-Salazar R., Zolezzi J.M., Inestrosa N.C. (2022). Inflammation context in Alzheimer’s disease, a relationship intricate to define. Biol. Res..

[96] Miao J., Ma H., Yang Y., Liao Y., Lin C., Zheng J., Yu M., Lan J. (2023). Microglia in Alzheimer’s disease: Pathogenesis, mechanisms, and therapeutic potentials. Front. Aging Neurosci..

[97] Zhang W., Xiao D., Mao Q., Xia H.R. (2023). Role of neuroinflammation in neurodegeneration development. Signal Transduct. Target. Ther..

[98] Lin M., Yu J.X., Zhang W.X., Lao F.X., Huang H.C. (2024). Roles of TREM2 in the Pathological Mechanism and the Therapeutic Strategies of Alzheimer’s Disease. J. Prev. Alzheimer’s Dis..

[99] Keren-Shaul H., Spinrad A., Weiner A., Matcovitch-Natan O., Dvir-Szternfeld R., Ulland T.K., David E., Baruch K., Lara-Astaiso D., Toth B. (2017). A unique microglia type associated with restricting development of Alzheimer’s disease. Cell.

[100] Zhao Y., Guo Q., Tian J., Liu W., Wang X. (2025). TREM2 bridges microglia and extracellular microenvironment: Mechanistic landscape and therapeutical prospects on Alzheimer’s disease. Ageing Res. Rev..

[101] Simpson D.S., Oliver P.L. (2020). ROS generation in microglia: Understanding oxidative stress and inflammation in neurodegenerative disease. Antioxidants.

[102] Wang X., Lopez O.L., Sweet R.A., Becker J.T., DeKosky S.T., Barmada M.M., Demirci F.Y., Kamboh M.I. (2014). Genetic determinants of disease progression in Alzheimer’s disease. J. Alzheimer’s Dis..

[103] Wang Y., Cella M., Mallinson K., Ulrich J.D., Young K.L., Robinette M.L., Gilfillan S., Krishnan G.M., Sudhakar S., Zinselmeyer B.H. (2015). TREM2 lipid sensing sustains the microglial response in an Alzheimer’s disease model. Cell.

[104] Atagi Y., Liu C.C., Painter M.M., Chen X.F., Verbeeck C., Zheng H., Li X., Rademakers R., Kang S.S., Xu H. (2015). Apolipoprotein E is a ligand for triggering receptor expressed on myeloid cells 2 (TREM2). J. Biol. Chem..

[105] Wolfe C.M., Fitz N.F., Nam K.N., Lefterov I., Koldamova R. (2018). The Role of APOE and TREM2 in Alzheimer’s Disease-Current Understanding and Perspectives. Int. J. Mol. Sci..

[106] Jendresen C., Årskog V., Daws M.R., Nilsson L.N. (2017). The Alzheimer’s disease risk factors apolipoprotein E and TREM2 are linked in a receptor signaling pathway. J. Neuroinflamm..

[107] Yeh F.L., Wang Y., Tom I., Gonzalez L.C., Sheng M. (2016). TREM2 Binds to Apolipoproteins, Including APOE and CLU/APOJ, and Thereby Facilitates Uptake of Amyloid-Beta by Microglia. Neuron.

[108] Song W., Hooli B., Mullin K., Jin S.C., Cella M., Ulland T.K., Wang Y., Tanzi R.E., Colonna M. (2017). Alzheimer’s disease-associated TREM2 variants exhibit either decreased or increased ligand-dependent activation. Alzheimer’s Dement..

[109] Shirotani K., Hori Y., Yoshizaki R., Higuchi E., Colonna M., Saito T., Hashimoto S., Saito T., Saido T.C., Iwata N. (2019). Aminophospholipids are signal-transducing TREM2 ligands on apoptotic cells. Sci. Rep..

[110] Kiialainen A., Hovanes K., Paloneva J., Kopra O., Peltonen L. (2005). Dap12 and Trem2, molecules involved in innate immunity and neurodegeneration, are co-expressed in the CNS. Neurobiol. Dis..

[111] Sirkis D.W., Bonham L.W., Aparicio R.E., Geier E.G., Ramos E.M., Wang Q., Karydas A., Miller Z.A., Miller B.L., Coppola G. (2016). Rare TREM2 variants associated with Alzheimer’s disease display reduced cell surface expression. Acta Neuropathol. Commun..

[112] Soragna D., Papi L., Ratti M.T., Sestini R., Tupler R., Montalbetti L. (2003). An Italian family affected by Nasu-Hakola disease with a novel genetic mutation in the TREM2 gene. J. Neurology. Neurosurg. Psychiatry.

[113] Sims R., van der Lee S.J., Naj A.C., Bellenguez C., Badarinarayan N., Jakobsdottir J., Kunkle B.W., Boland A., Raybould R., Bis J.C. (2017). Rare coding variants in PLCG2, ABI3, and TREM2 implicate microglial-mediated innate immunity in Alzheimer’s disease. Nat. Genet..

[114] Jin S.C., Benitez B.A., Karch C.M., Cooper B., Skorupa T., Carrell D., Norton J.B., Hsu S., Harari O., Cai Y. (2014). Coding variants in TREM2 increase risk for Alzheimer’s disease. Hum. Mol. Genet..

[115] Jiang T., Tan L., Chen Q., Tan M.S., Zhou J.S., Zhu X.C., Lu H., Wang H.F., Zhang Y.D., Yu J.T. (2016). A rare coding variant in TREM2 increases risk for Alzheimer’s disease in Han Chinese. Neurobiol. Aging.

[116] Jiang T., Hou J.K., Gao Q., Yu J.T., Zhou J.S., Zhao H.D., Zhang Y.D. (2016). TREM2 p. H157Y variant and the risk of Alzheimer’s disease: A meta-analysis involving 14,510 subjects. Curr. Neurovascular Res..

[117] Jin S.C., Carrasquillo M.M., Benitez B.A., Skorupa T., Carrell D., Patel D., Lincoln S., Krishnan S., Kachadoorian M., Reitz C. (2015). *TR*EM2 is associated with increased risk for Alzheimer’s disease in African Americans. Mol. Neurodegener..

[118] Jiang T., Carrasquillo M.M., Benitez B.A., Skorupa T., Carrell D., Patel D., Lincoln S., Krishnan S., Kachadoorian M., Reitz C. (2013). TREM2 in Alzheimer’s disease. Mol. Neurobiol..

[119] Ulrich J.D., Ulland T.K., Colonna M., Holtzman D.M. (2017). Elucidating the Role of TREM2 in Alzheimer’s Disease. Neuron.

[120] Song W.M., Joshita S., Zhou Y., Ulland T.K., Gilfillan S., Colonna M. (2018). Humanized TREM2 mice reveal microglia-intrinsic and-extrinsic effects of R47H polymorphism. J. Exp. Med..

[121] Dodd R.B. (2018). An exTREMe disruption in Alzheimer’s cleanup. J. Biol. Chem..

[122] Gratuze M., Leyns C.E., Sauerbeck A.D., St-Pierre M.K., Xiong M., Kim N., Serrano J.R., Tremblay M.È., Kummer T.T., Colonna M. (2020). Impact of TREM2 R47H variant on tau pathology–induced gliosis and neurodegeneration. J. Clin. Investig..

[123] Hall-Roberts H., Agarwal D., Obst J., Smith T.B., Monzón-Sandoval J., Di Daniel E., Webber C., James W.S., Mead E., Davis J.B. (2020). TREM2 Alzheimer’s variant R47H causes similar transcriptional dysregulation to knockout, yet only subtle functional phenotypes in human iPSC-derived macrophages. Alzheimer’s Res. Ther..

[124] Park J.-S., Ji I.J., Kim D.H., An H.J., Yoon S.Y. (2017). The Alzheimer’s disease-associated R47H variant of TREM2 has an altered glycosylation pattern and protein stability. Front. Neurosci..

[125] Zhu B., Liu Y., Hwang S., Archuleta K., Huang H., Campos A., Murad R., Piña-Crespo J., Xu H., Huang T.Y. (2022). Trem2 deletion enhances tau dispersion and pathology through microglia exosomes. Mol. Neurodegener..

[126] Jain N., Lewis C.A., Ulrich J.D., Holtzman D.M. (2023). Chronic TREM2 activation exacerbates Aβ-associated tau seeding and spreading. J. Exp. Med..

[127] Huang W., Huang J., Huang N., Luo Y. (2023). The role of TREM2 in Alzheimer’s disease: From the perspective of Tau. Front. Cell Dev. Biol..

[128] Zhou Y., Song W.M., Andhey P.S., Swain A., Levy T., Miller K.R., Poliani P.L., Cominelli M., Grover S., Gilfillan S. (2020). Human and mouse single-nucleus transcriptomics reveal TREM2-dependent and TREM2-independent cellular responses in Alzheimer’s disease. Nat. Med..

[129] Xiang X., Kawauchi S., Kramár E.A., Rezaie N., Liang H.Y., Sakr J.S., Gomez-Arboledas A., Arreola M.A., Cunha C.D., Phan J. (2018). The Trem2 R47H Alzheimer’s risk variant impairs splicing and reduces Trem2 mRNA and protein in mice but not in humans. Mol. Neurodegener..

[130] Cheng-Hathaway P.J., Reed-Geaghan E.G., Jay T.R., Casali B.T., Bemiller S.M., Puntambekar S.S., von Saucken V.E., Williams R.Y., Karlo J.C., Moutinho M. (2018). The T rem 2 R47H variant confers loss-of-function-like phenotypes in Alzheimer’s disease. Mol. Neurodegener..

[131] Budyak E.I., Kwon J., Messenger E.J., Maharjan S., Koothur J.J. (2023). TREM2 Alteration Increases AD Biomarkers and is Associated with Key Genes with 5xFAD Mice Model Analysis on MODEL-AD Database. bioRxiv.

[132] Menzies G.E., Sims R., Williams J. (2019). Molecular Dynamics simulations of Alzheimer’s variants, R47H and R62H, in TREM2 provide evidence for structural alterations behind functional changes. bioRxiv.

[133] Kober D.L., Alexander-Brett J.M., Karch C.M., Cruchaga C., Colonna M., Holtzman M.J., Brett T.J. (2016). Neurodegenerative disease mutations in TREM2 reveal a functional surface and distinct loss-of-function mechanisms. Elife.

[134] Krasemann S., Madore C., Cialic R., Baufeld C., Calcagno N., El Fatimy R., Beckers L., O’Loughlin E., Xu Y., Fanek Z. (2017). The TREM2-APOE Pathway Drives the Transcriptional Phenotype of Dysfunctional Microglia in Neurodegenerative Diseases. Immunity.

[135] Colonna M. (2023). The biology of TREM receptors. Nat. Rev. Immunol..

[136] Li R., Wang X., He P. (2021). The most prevalent rare coding variants of TREM2 conferring risk of Alzheimer’s disease: A systematic review and meta-analysis. Exp. Ther. Med..

[137] Carmona S., Zahs K., Wu E., Dakin K., Bras J., Guerreiro R. (2018). The role of TREM2 in Alzheimer’s disease and other neurodegenerative disorders. Lancet Neurol..

[138] Zhao Y., Li X., Huang T., Jiang L.L., Tan Z., Zhang M., Cheng I.H., Wang X., Bu G., Zhang Y.W. (2017). Intracellular trafficking of TREM2 is regulated by presenilin 1. Exp. Mol. Med..

[139] Park J.S., Ji I.J., An H.J., Kang M.J., Kang S.W., Kim D.H., Yoon S.Y. (2015). Disease-associated mutations of TREM2 alter the processing of N-linked oligosaccharides in the Golgi apparatus. Traffic.

[140] Joshi P., Riffel F., Satoh K., Enomoto M., Qamar S., Scheiblich H., Villacampa N., Kumar S., Theil S., Parhizkar S. (2021). Differential interaction with TREM2 modulates microglial uptake of modified Aβ species. Glia.

[141] Sudom A., Talreja S., Danao J., Bragg E., Kegel R., Min X., Richardson J., Zhang Z., Sharkov N., Marcora E. (2018). Molecular basis for the loss-of-function effects of the Alzheimer’s disease–associated R47H variant of the immune receptor TREM2. J. Biol. Chem..

[142] Kleinberger G., Yamanishi Y., Suárez-Calvet M., Czirr E., Lohmann E., Cuyvers E., Struyfs H., Pettkus N., Wenninger-Weinzierl A., Mazaheri F. (2014). TREM2 mutations implicated in neurodegeneration impair cell surface transport and phagocytosis. Sci. Transl. Med..

[143] Kleinberger G., Brendel M., Mracsko E., Wefers B., Groeneweg L., Xiang X., Focke C., Deußing M., Suárez-Calvet M., Mazaheri F. (2017). The FTD-like syndrome causing TREM2 T66M mutation impairs microglia function, brain perfusion, and glucose metabolism. EMBO J..

[144] Shi Q., Gutierrez R.A., Bhat M.A. (2025). Microglia, Trem2, and Neurodegeneration. Neuroscientist.

[145] Gao CJiang J., Tan Y., Chen S. (2023). Microglia in neurodegenerative diseases: Mechanism and potential therapeutic targets. Signal Transduct. Target. Ther..

[146] Dash R., Choi H.J., Moon I.S. (2020). Mechanistic insights into the deleterious roles of Nasu-Hakola disease associated TREM2 variants. Sci. Rep..

[147] Sirkis D.W., Aparicio R.E., Schekman R. (2017). Neurodegeneration-associated mutant TREM2 proteins abortively cycle between the ER and ER-Golgi intermediate compartment. Mol. Biol. Cell.

[148] Plotkin L.I. (2019). Triggering Receptor Expressed on Myeloid Cells 2 (TREM2) Mutations: A Potential Common Cause of Alzheimer’s Disease and Musculoskeletal Disorders. FASEB J..

[149] Schlepckow K., Kleinberger G., Fukumori A., Feederle R., Lichtenthaler S.F., Steiner H., Haass C. (2017). An Alzheimer-associated TREM2 variant occurs at the ADAM cleavage site and affects shedding and phagocytic function. EMBO Mol. Med..

[150] Qiao W., Chen Y., Zhong J., Madden B.J., Charlesworth C.M., Martens Y.A., Liu C.C., Knight J., Ikezu T.C., Kurti A. (2023). Trem2 H157Y increases soluble TREM2 production and reduces amyloid pathology. Mol. Neurodegener..

[151] Miyashita A., Wen Y., Kitamura N., Matsubara E., Kawarabayashi T., Shoji M., Tomita N., Furukawa K., Arai H., Asada T. (2014). Lack of genetic association between TREM2 and late-onset Alzheimer’s disease in a Japanese population. J. Alzheimer’s Dis. JAD.

[152] Feuerbach D., Schindler P., Barske C., Joller S., Beng-Louka E., Worringer K.A., Kommineni S., Kaykas A., Ho D.J., Ye C. (2017). ADAM17 is the main sheddase for the generation of human triggering receptor expressed in myeloid cells (hTREM2) ectodomain and cleaves TREM2 after Histidine 157. Neurosci Lett..

[153] Thornton P., Sevalle J., Deery M.J., Fraser G., Zhou Y., Ståhl S., Franssen E.H., Dodd R.B., Qamar S., Gomez Perez-Nievas B. (2017). TREM2 shedding by cleavage at the H157-S158 bond is accelerated for the Alzheimer’s disease-associated H157Y variant. EMBO Mol. Med..

[154] Patel D., Mez J., Vardarajan B.N., Staley L., Chung J., Zhang X., Farrell J.J., Rynkiewicz M.J., Cannon-Albright L.A., Teerlink C.C. (2019). Association of Rare Coding Mutations with Alzheimer Disease and Other Dementias Among Adults of European Ancestry. JAMA Netw. Open.

[155] Paloneva J., Autti T., Hakola P., Haltia M.J. (1993). Polycystic Lipomembranous Osteodysplasia with Sclerosing Leukoencephalopathy (PLOSL). GeneReviews (^®^).

[156] Ji M.J., Jung S., Seo H.E., Kim S.Y., Kim W.R., Kim S., Lee J.S., Noh Y. (2020). Heterozygous TREM2 Mutation in Semantic Variant of Primary Progressive Aphasia. J. Clin. Neurol..

[157] Haddad G. (2024). Unraveling the Role of TREM2 and CD33 in Alzheimer’s Disease. Am. J. Stud. Res..

[158] Suárez-Calvet M., Morenas-Rodríguez E., Kleinberger G., Schlepckow K., Araque Caballero M.Á., Franzmeier N., Capell A., Fellerer K., Nuscher B., Eren E. (2019). Early increase of CSF sTREM2 in Alzheimer’s disease is associated with tau related-neurodegeneration but not with amyloid-β pathology. Mol. Neurodegener..

[159] Tian Y., Xiao X., Liu W., Cheng S., Qian N., Wang L., Liu Y., Ai R., Zhu X. (2024). TREM2 improves microglia function and synaptic development in autism spectrum disorders by regulating P38 MAPK signaling pathway. Mol. Brain.

[160] George J. (2023). TREM2 as an evolving therapeutic target in Alzheimer’s disease. Neural Regen. Res..

[161] Fassler M., Rappaport M.S., Cuño C.B., George J. (2021). Engagement of TREM2 by a novel monoclonal antibody induces activation of microglia and improves cognitive function in Alzheimer’s disease models. J. Neuroinflamm..

[162] Zhang L., Xiang X., Li Y., Bu G., Chen X.F. (2025). TREM2 and sTREM2 in Alzheimer’s disease: From mechanisms to therapies. Mol. Neurodegener..

[163] Mirescu C. (2025). Characterization of the first TREM2 small molecule agonist, VG-3927, for clinical development in Alzheimer’s disease. Alzheimers Dement..

[164] Vigil’s TREM2-Targeted Alzheimer’s Treatment Shows Early Promise, Moves on to Phase II. https://www.biospace.com/drug-development/vigils-trem2-targeted-alzheimers-treatment-shows-early-promise-moves-on-to-phase-ii.

[165] Ma Y.N., Hu X., Karako K., Song P., Tang W., Xia Y. (2025). The potential and challenges of TREM2-targeted therapy in Alzheimer’s disease: Insights from the INVOKE-2 study. Front. Aging Neurosci..

[166] Colonna M., Holtzman D.M. (2025). Rethinking TREM2 as a target for Alzheimer’s disease after the INVOKE-2 trial failure. Nat. Med..

[167] Serradas M.L., Ding Y., Martorell P.V., Kulińska I., Castro-Gomez S. (2024). Therapeutic Targets in Innate Immunity to Tackle Alzheimer’s Disease. Cells.

[168] van Lengerich B., Zhan L., Xia D., Chan D., Joy D., Park J.I., Tatarakis D., Calvert M., Hummel S., Lianoglou S. (2023). A TREM2-activating antibody with a blood-brain barrier transport vehicle enhances microglial metabolism in Alzheimer’s disease models. Nat. Neurosci..

[169] Schlepckow K., Monroe K.M., Kleinberger G., Cantuti-Castelvetri L., Parhizkar S., Xia D., Willem M., Werner G., Pettkus N., Brunner B. (2020). Enhancing protective microglial activities with a dual function TREM2 antibody to the stalk region. EMBO Mol. Med..

[170] Zhao P., Xu Y., Jiang L., Fan X., Li L., Li X., Arase H., Zhao Y., Cao W., Zheng H. (2022). A tetravalent TREM2 agonistic antibody reduced amyloid pathology in a mouse model of Alzheimer’s disease. Sci. Transl. Med..

[171] Masoudi N., Willen J., Daniels C., Jenkins B.A., Furber E.C., Kothiya M., Banjoko M.B., Gowda R., Hendricks J., Fang Y.Y. (2024). Microglial-targeted gene therapy: Developing a disease modifying treatment for ALSP associated with CSF1R Mutations (ALSP-CSF1R) (P11-4.012). Neurology.

[172] Yoo Y., Neumayer G., Shibuya Y., Mader M.M., Wernig M. (2023). A cell therapy approach to restore microglial Trem2 function in a mouse model of Alzheimer’s disease. Cell Stem Cell..

[173] Deming Y., Li Z., Benitez B.A., Cruchaga C. (2018). Triggering receptor expressed on myeloid cells 2 (TREM2): A potential therapeutic target for Alzheimer disease?. Expert. Opin. Ther. Targets.

[174] Guo H., Wang M., Ni C., Yang C., Fu C., Zhang X., Chen X., Wu X., Hou J., Wang L. (2025). TREM2 promotes the formation of a tumor-supportive microenvironment in hepatocellular carcinoma. J. Exp. Clin. Cancer Res..

